# What Is Long-Term Survival and Which First-Line Immunotherapy Brings Long-Term Survival for Advanced Wild-Type Non-Small Cell Lung Cancer: A Network Meta-Analysis Based on Integrated Analysis

**DOI:** 10.3389/fimmu.2022.764643

**Published:** 2022-04-05

**Authors:** Xue Zhang, Qian Xu, Xuejun Yu, Miao Huang, Song Li, Lei Sheng, Xin Dai, Kai Huang, Jian Wang, Lian Liu

**Affiliations:** Department of Medical Oncology, Qilu Hospital, Cheeloo College of Medicine, Shandong University, Jinan, China

**Keywords:** long-term survival, first-line, immunotherapy, non-small cell lung cancer, network meta-analysis, integrated analysis

## Abstract

**Background:**

Immune checkpoint inhibitors (ICIs) have significantly improved survival for advanced wild-type non-small cell lung cancer, but there is no direct comparison to confirm which first-line treatment may lead to the longest overall survival. What qualifies as long-term survival (LS) is even unclear.

**Methods:**

By searching PubMed, Embase, and the Cochrane Central Register of Controlled Trials from January 2005 to December 2020, we included randomized controlled trials (RCTs) of first-line ICI-containing treatments to perform an integrated analysis (IA) to determine the criterion of LS and then screened regimens with LS for network meta-analysis (NMA). The main outcomes for NMA were median overall survival (mOS), 1-year survival rate (1ySR), and 2-year survival rate (2ySR); those for IA were the pooled mOS (POS), 1ySR (P1SR), and 2ySR (P2SR).

**Results:**

By IA of 16 first-line ICIs from 20 RCTs, the POS was 16.20 (95% CI 14.79–17.60) months, with P1SR of 63% (95% CI 59–66%) and P2SR of 37% (33–41%). Thus, we defined LS as mOS ≥ POS (16.20 m) for regimens and screened for RCTs with outcomes meeting this criterion. Eleven ICI-based regimens can bring LS for the overall population, among which ICI with bevacizumab and chemotherapy achieved the longest POS of 19.50 m (16.90–22.10 m) and the highest P1SR (74%, 61%–87%) and P2SR (49%, 38%–61%). Pembrolizumab with chemotherapy ranked first in mOS and 1ySR, while atezolizumab plus bevacizumab and chemotherapy ranked first in 2ySR.

**Conclusions:**

Through the IA of first-line treatment regimens, a POS of 16.20 m can be determined as the LS standard. Further considering 1ySR and 2ySR, atezolizumab combined with bevacizumab and chemotherapy or pembrolizumab plus chemotherapy are likely to bring the longest LS in the overall population, while single ICI may be adequate for patients with a high PD-L1 expression. ICIs with bevacizumab and chemotherapy may be the best combination for LS for its further advantage over time.

## Introduction

In recent years, the development and application of immune checkpoint inhibitors (ICIs), including programmed cell death protein 1 (PD-1) antibody, programmed death ligand 1 (PD-L1) antibody, and cytotoxic T-lymphocyte-associated protein 4 (CTLA-4) antibody, have provided significant survival benefits for patients with wild-type advanced NSCLC. In the successful randomized controlled trials (RCTs), such as KEYNOTE189 (1), KEYNOTE407 (2), IMpower110 (3), IMpower130 (4), IMpower132 (5), and CheckMate227 (6, 7), immuno-related therapy can prolong the mOS of advanced wild-type NSCLC to 17~22 months. The 5-year survival rate in the KEYNOTE024 study with PD-L1 ≥50% NSCLC increased to 31.9% in the PEM monotherapy group (8). The unprecedented long-term survivals of patients with immunotherapy are determined by the characteristics of the immune response process. Tumor cells recognized and killed by activated T lymphocytes will further release antigens to activate more T lymphocytes, thus forming a positive cycle of the self-activation process, during which immune memory cells are also generated (9). The positive feedback promotes the immune-active T cells to function for a long time, thus bringing a longer survival to these patients.

At present, it is widely accepted that immunotherapy can bring long-term survival to patients with advanced wild-type NSCLC. In fact, large differences existed in OS among different ICIs [11.2 (10) ~26.3 months (8)]. Therefore, we can hardly judge how long in general the first-line immuno-related treatment can bring to advanced NSCLC patients. Neither do we know whether there is a difference in efficacy among the treatments that have achieved significant OS benefits and are recommended by kinds of guidelines, or in other words, which regimen can lead to the longest survival. In fact, for immunotherapy, how to define long-term survival (LS) is an open question.

In order to answer these questions, we conducted an integrated analysis of the survival outcomes of RCTs on first-line immuno-related therapies. Pooled median OS (POS) was taken as the cutoff value for LS to screen the treatment regimens that can bring LS. Then, a network meta-analysis (NMA) based on the Bayesian model was performed to compare and rank these treatment regimens with LS according to mOS, 1-year survival rate (1ySR), 2-year survival rate (2ySR), and other efficacy and adverse effects (AEs) in the general population and special population. Our goal was to provide valuable information for clinicians looking for the best first-line treatment for patients with advanced driver gene-negative NSCLC.

## Materials and Methods

This NMA was performed in accordance with the PRISMA extension statement for NMA (**Supplementary Table 1**) (11). The research was registered with PROSPERO (CRD42020184534).

### Data Sources and Searches

PubMed, Embase, the Cochrane Central Register of Controlled Trials, and ClinicalTrials.gov databases were searched to find relevant articles from January 2005 to December 2020. Abstracts on NSCLC from several important international conferences (American Society of Clinical Oncology, ESMO, and World Conference on Lung Cancer) from 2015 to 2020 were inspected to identify potentially relevant studies. For an outcome in the same trial, only the most recent data were kept. The detailed search strategy is shown in **Supplementary Table 2**.

### Eligibility Criteria

We included published phase II/III RCTs reported in English. The mOS integrated analysis enrolled previously untreated patients with advanced (stage III/IV or recurrent) histologically confirmed wild-type NSCLC, treated with ICI-containing regimens, and the mOS or 1- or 2-year survival rate with a 95% confidence interval (CI) was available. According to the POS criteria, RCTs meeting the needs of LS were screened for NMA analysis. mOS of subgroups such as high PD-L1 expression or tumor mutation burden (TMB) that met the LS criteria was included in subgroup analysis. Large RCTs with chemotherapy (CT) combined with anti-angiogenesis therapy (AA) were included in the network as controls. Exclusion criteria included targeted therapy for advanced NSCLC with positive driver genes, trials including radiotherapy, cell therapy, vaccine, heat shock protein, and other non-ICI-related therapy.

### Data Extraction and Risk-of-Bias Assessment

The bias risk of included trials was assessed using the Cochrane risk-of-bias tool, consisting of random sequence generation, allocation concealment, blinding of participants and personnel, blinding of outcome assessment, incomplete outcome data, selective outcome reporting, and other sources of bias (12) (**Supplementary Figure 1**). We extracted detailed clinical trial data (e.g., study ID, first author, year of publication, number of patients, patient characteristics), treatments, and outcomes into a spreadsheet. Data extraction and quality assessment were conducted independently by the two authors (ZX and XQ), and any differences were resolved through discussion and negotiation.

### Data Synthesis and Analysis

First, the mOS of first-line immuno-related therapy in advanced wild-type NSCLC was analyzed using STATA 16.0 to determine LS criteria. We also pooled the 1ySR and 2ySR using STATA 16.0 (13). For PFS and OS in NMA, the hazard ratio (HR) and its 95% creditable intervals (CrIs) were calculated, while for binary variables, such as 1ySR and 2ySR, 1-year progression-free survival rate (1yPR), overall response rate (ORR), ≥3 AEs, odds ratio (OR) and 95% CrIs were calculated. For some indexes, some of the studies (5 (14–18) of 15 for the 1-year OS rate, 5 (3, 7, 14–16) of 13 for the 2-year OS rate, 6 (3, 14–17, 19) of 14 for the 1-year PFS rate) did not provide ORs for which we got them from the Kaplan–Meier curve and calculated them by STATA 16.0. All network evidence maps were obtained by STATA16.0 (20). The NMA was performed in a Bayesian framework using a Markov chain Monte Carlo simulation technique within the GEMTC package in the R-Statistics and the J.A.G.S. program. We used non-informative uniform and normal prior distributions to fit the model, with four different sets of initial values. For each outcome, 150,000 sample iterations were generated with 100,000 burn-ins and a thinning interval of 1 except for ORR and ≥3 AEs, for which we increased the thinning interval to 10 to minimize auto-correlation. The convergence of the model is evaluated by a diagnostic convergence graph and a trace density graph (21). Fixed- and random-effect models were considered and compared using deviance information criteria (DIC). If the DIC difference between the random model and the fixed model was less than 5, the fixed model was selected. A direct and indirect comparison of inconsistency analysis was verified by DIC and node analysis (22). Preferred probability ranking was obtained from the surface under the cumulative ranking curve (SUCRA). The line charts for the rankings were produced using GraphPad Prism 8.

## Results

### Qualified Studies for Integrated Analysis

**Figure 1:**
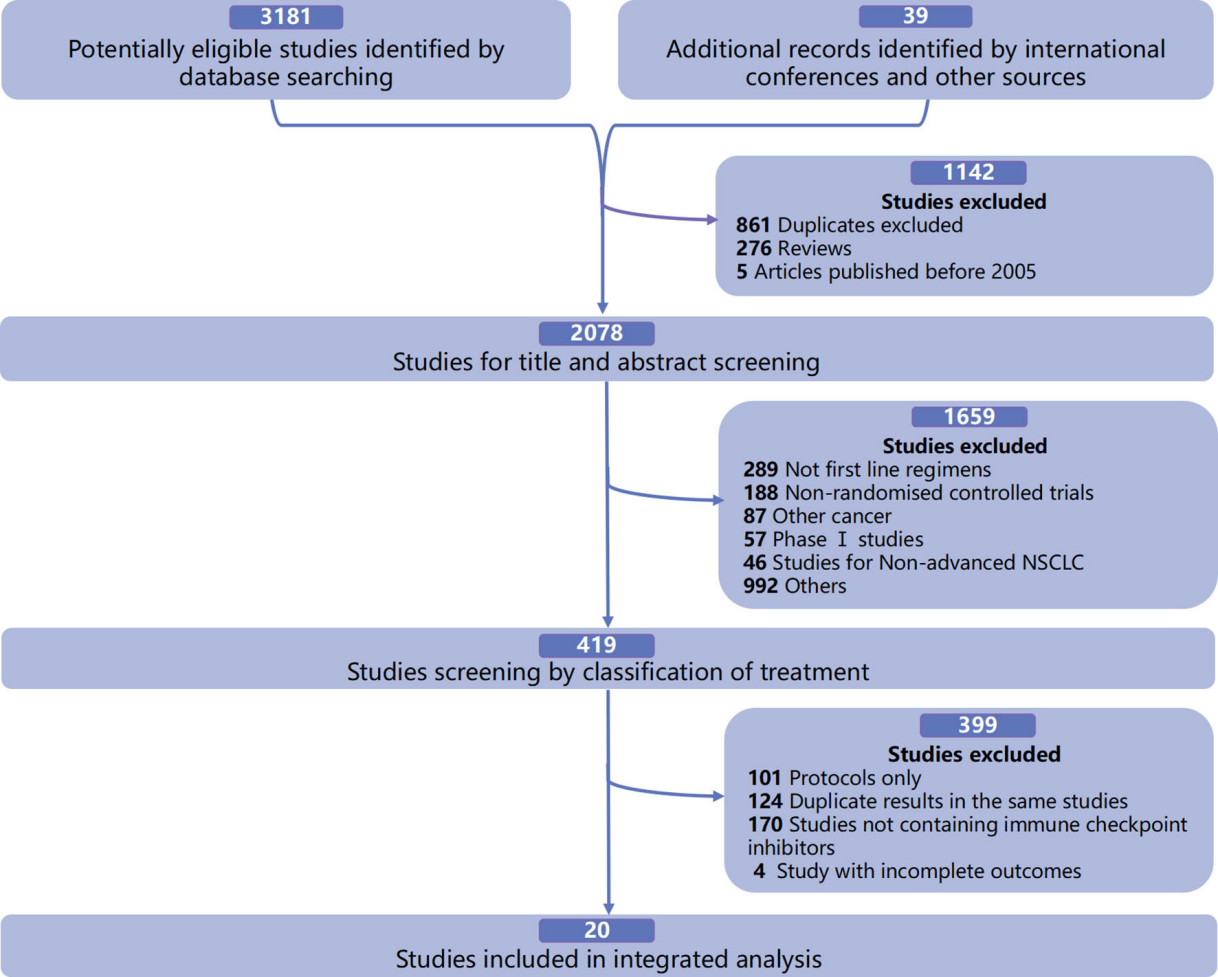
Study selection.

In the integrated analysis of the available mOS and survival rates of first-line immuno-related treatments in patients with advanced driver gene-negative NSCLC, a total of 20 qualified RCTs were included (**Figure 1**), involving 7,462 patients and 16 treatment regimens, including single ICIs such as pembrolizumab (PEM) (8, 18), atezolizumab (ATE) (3), nivolumab (NIV) (23), durvalumab (DUR) (10), and cemiplimab (CEM) (24); dual ICIs such as ipilimumab (IPI) combined with NIV (NIV+IPI) (6) and tremelimumab (TRE) combined with DUR (DUR+TRE) (10); ICIs combined with CT such as PEM+CT (1, 2, 19), ATE+CT (4, 5, 25, 26), NIV+CT (7), IPI+CT (27, 28), camrelizumab (CAM) combined with CT (CAM+CT) (17), NIV+IPI+CT (29), and DUR+TRE+CT (16); and ICIs combined with AA and CT, such as ATE combined with bevacizumab (BEV) and CT (ATE+BEV+CT) (26), and NIV+BEV+CT (30) (**Supplementary Table 3**).

### Integrated Analysis

**Figure 2:**
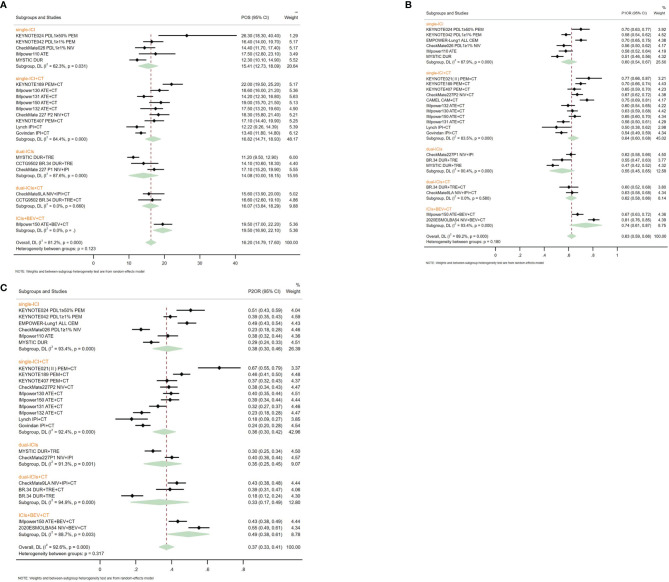
Pooled survival outcomes from integrated analysis of median overall survival **(A)**, 1-year survival rate **(B)**, and 2-year survival rate **(C)** of different therapy strategies containing immune checkpoint inhibitors in patients with advanced wild-type non-small cell lung cancer.

**Figure 3:**
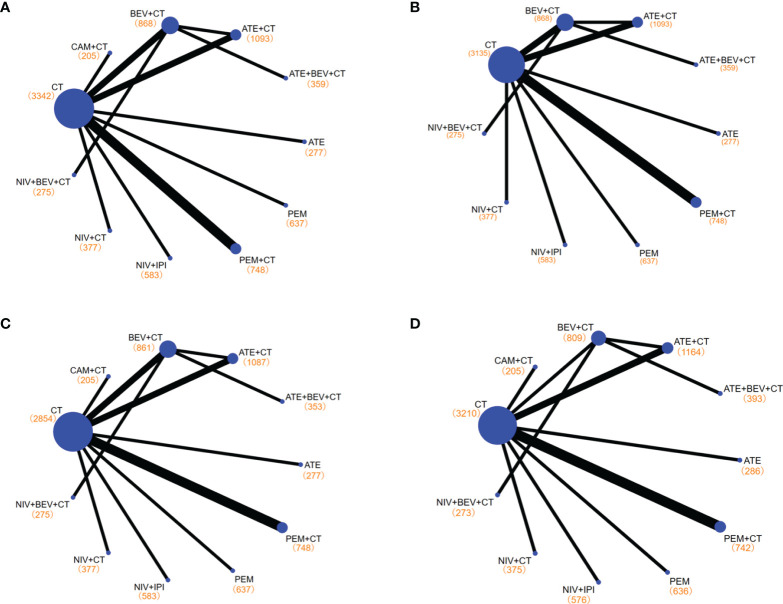
Network diagrams of comparisons on different outcomes of treatments with long-term survival time in patients with advanced wild-type non-small cell lung cancer. **(A)** Comparisons on overall survival (OS) and progression-free survival (PFS) and 1-year survival rate and 1-year PFS rate. **(B)** Comparisons on 2-year survival rate. **(C)** Comparisons on objective response rate (ORR). **(D)** Comparisons on adverse events of grade 3 or higher (≥3 AEs). Each circular node represents a type of treatment. Each line represents a type of head-to-head comparison. The size of the nodes and the thickness of the lines are weighted according to the number of studies evaluating each treatment and direct comparison, respectively. The total number of patients receiving a treatment is shown in brackets. PEM, pembrolizumab; ATE, atezolizumab; NIV, nivolumab; CAM, camrelizumab; BEV, bevacizumab; CEM, cemiplimab; CT, chemotherapy.

First, we integrated the available mOS of 13 first-line immuno-related treatments in wild-type patients (**Figure 2A**) and found that POS was 16.20 (95% CI 14.79~17.60) months. Therefore, we set mOS over 16.20 months as the standard for LS for immunotherapy. In the ITT population, there were 11 treatments that met the criteria for long-term survival (**Figure 3**), most of which were ICIs combined with CT and/or AA, including PEM+CT, NIV+CT, ATE+CT, CAM+CT, DUR+TRE+CT, ATE+BEV+CT, and NIV+BEV+CT, and single ICI (PEM, ATE) and dual ICIs (NIV+IPI, DUR+ TRE) (**Supplementary Table 4**). In subgroups with PD-L1 ≥50%, treatments leading to LS included single ICIs (CEM, PEM, ATE, DUR, NIV), ICIs combined with CT (PEM+CT, ATE+CT), and dual ICIs (NIV+IPI) or in combination of CT (NIV+IPI+CT) (**Supplementary Table 5**). Among the TMB-high subgroups, single ICIs (PEM, NIV, ATE, DUR) or immune combination regimens (PEM+CT, NIV+IPI, TRE+DUR, DUR+TRE+CT) can bring LS.

Among the various treatment strategies, ICIs+BEV+CT led to the longest POS (19.50, 16.90~22.10 months), followed by single ICIs+CT (16.82, 14.71~18.93 months) and dual ICIs+CT (16.07 months, 13.84~18.29). Single ICIs (15.41 months, 12.73~18.09) and dual ICIs (14.08 months, 10.00~18.15) had the shortest POS (**Figure 2A**). Comparing the different ICI targets, the anti-PD-1-containing regimens had a maximum POS of 18.00 (15.52~20.48) months, followed by the anti-PD-L1 regimens of 17.23 (15.17~19.30) months. Comparing different ICIs, the regimens containing PEM had the longest POS of 19.09 (15.69~22.48) months, while the IPI-containing regimens had the shortest POS of 13.10 (11.80~14.39) months (**Supplementary Table 6**).

In the integrated analysis of 1ySR, a total of 20 RCTs were included, involving 16 regimens, including single ICIs [PEM (8, 18), ATE (3), CEM (24), NIV (23), DUR (10)], dual ICIs [NIV+IPI (6), DUR+TRE (10)], ICIs+CT [PEM+CT (1, 2, 19), ATE+CT (4, 5, 25, 26), NIV+CT (7), CAM+CT (17), IPI+CT (27, 28), NIV+IPI+CT (29), DUR+TRE+CT (16)], and ICIs+BEV+CT [NIV+BEV+CT (30), ATE+BEV+CT (26)]. The P1SR was 0.63 (0.59~0.66). Among them, the P1SR of single ICIs, dual ICIs, single ICIs+CT, dual ICIs+CT, and ICIs+BEV+CT were 0.60 (0.54~0.67), 0.55 (0.45~0.65), 0.64 (0.60~0.68), 0.62 (0.58~0.66), and 0.74 (0.67~0.87), respectively (**Figure 2B**). In the integrated analysis of 19 RCTs with LS (1–8, 10, 16, 18, 19, 23–30), we found the P2SR to be 0.37 (0.33~0.41). ICIs+BEV+CT brought the highest P2SR (0.49, 0.38~0.61), and single ICIs, dual ICIs, single ICIs+CT, and dual ICIs+CT got 0.38 (0.30~0.46), 0.35 (0.25~0.45), 0.36 (0.30~0.42), and 0.33 (0.17~0.49), respectively (**Figure 2C**).

In terms of pathological types, non-squamous cell lung carcinoma had a POS of 19.32 (18.12~20.51) months in first-line ICIs regimens, longer than that of 15.13 (13.39~16.88) months in squamous cell lung carcinoma patients. The P1SR and P2SR were 0.69 (0.64~0.73) and 0.44 (0.37~0.50) in the non-squamous group, and 0.60 (0.55~0.66) and 0.33 (0.27~0.38) in the squamous subgroup, respectively (**Supplementary Figures 2A, B**).

In the population with PD-L1 ≥50%, the P1SR and P2SR induced by the first-line immunotherapy were 0.67 (0.65~0.69) and 0.46 (0.44~0.48), respectively (**Supplementary Figure 2C**). When comparing regimens, similar to the ITT population, the combination of single ICIs with AA and CT seemed to bring the highest P1SR (0.75,0.65~0.85) and P2SR (0.56, 0.45~0.67). The P2SR was similar between the single ICIs (0.47, 0.44~0.50) and the dual ICIs (0.48, 0.41~0.55). Moreover, the addition of CT to single ICIs or dual ICIs failed to improve the P2SR over ICIs alone (**Supplementary Table 6**).

### NMA for the ITT Population

An assessment of the risk bias in the included studies is shown in **Supplementary Figure S2**. It should be noted that some treatment options that only met the LS criteria in the corresponding subgroup were included in the subgroup analysis. For example, NIV+IPI+CT of CheckMate 9LA was only included in the subgroup analysis for PD-L1 ≥50% (29), while BEV+CT and CT alone were included as controls in NMA (14, 15).

**Figure 4:**
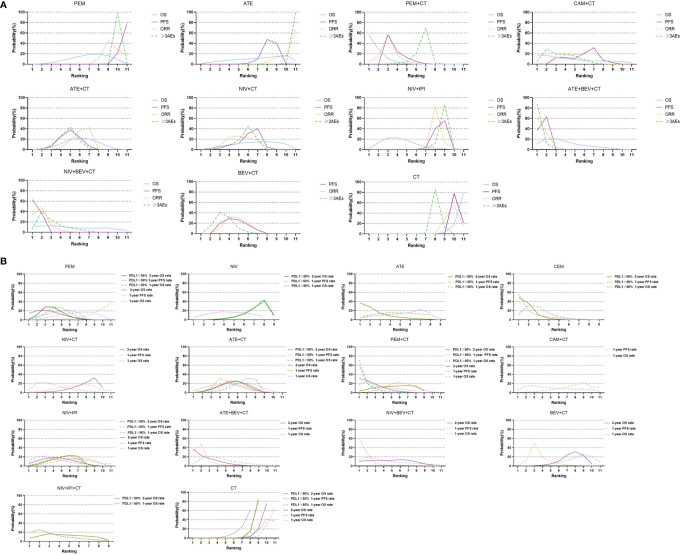
Bayesian ranking profiles of comparable treatments with long-term survival on efficacy and safety for patients with advanced NSCLC. **(A)** Profiles indicate the probability of each comparable treatment being ranked from first to last on overall survival (OS), progression-free survival (PFS), objective response rate (ORR), and grade 3 or higher adverse events (≥3 AEs). **(B)** Profiles indicate the probability of each comparable treatment being ranked from first to last on 1-year survival rate and 1-year PFS rate and 2-year survival rate in the overall population, and 1-year survival rate and 1-year PFS rate and 2-year survival rate in patients with high PD-L1 subgroups. PEM, pembrolizumab; ATE, atezolizumab; NIV, nivolumab; DUR, durvalumab; TRE, tremelimumab; IPI, ipilimumab; CAM, camrelizumab; CEM, cemiplimab; BEV, bevacizumab; CT, chemotherapy.

**Figure 5:**
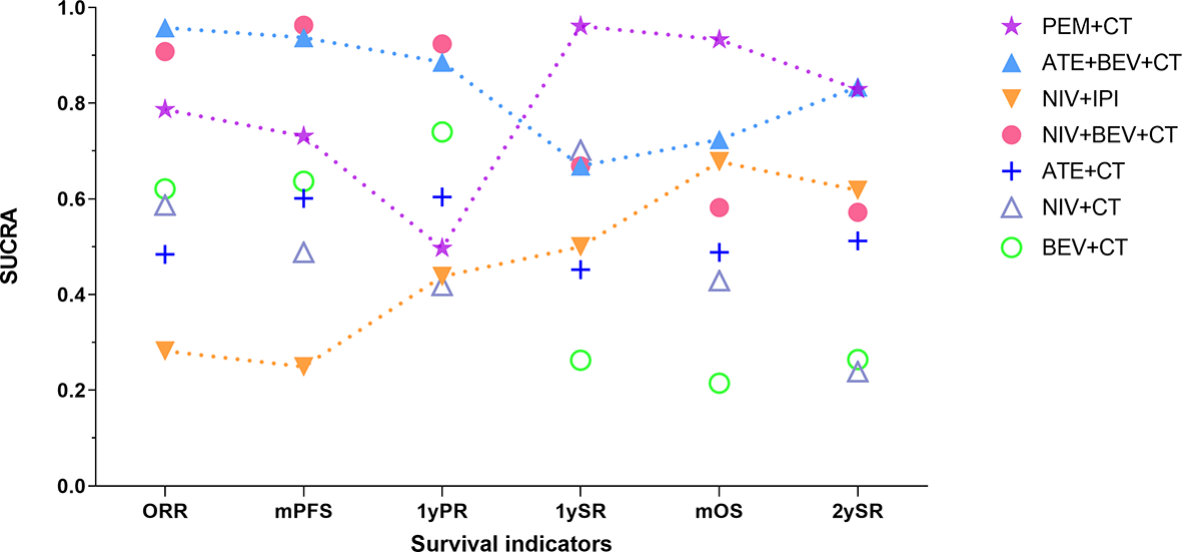
Changing tendency in efficacy ranking of main immuno-related therapies for advanced wild-type NSCLC as time goes on. The vertical axis represents the SUCRA value of ranking probability, and the horizontal axis represents survival indicators arranged in chronological order. PEM, pembrolizumab; ATE, atezolizumab; NIV, nivolumab; IPI, ipilimumab; BEV, bevacizumab; CT, chemotherapy; ORR, objective response rate; mPFS, median progression-free survival time; 1yPR, 1-year PFS rate, mOS, median overall survival time; 1ySR, 1-year survival rate; 2ySR, 2-year survival rate.

**Figure 6:**
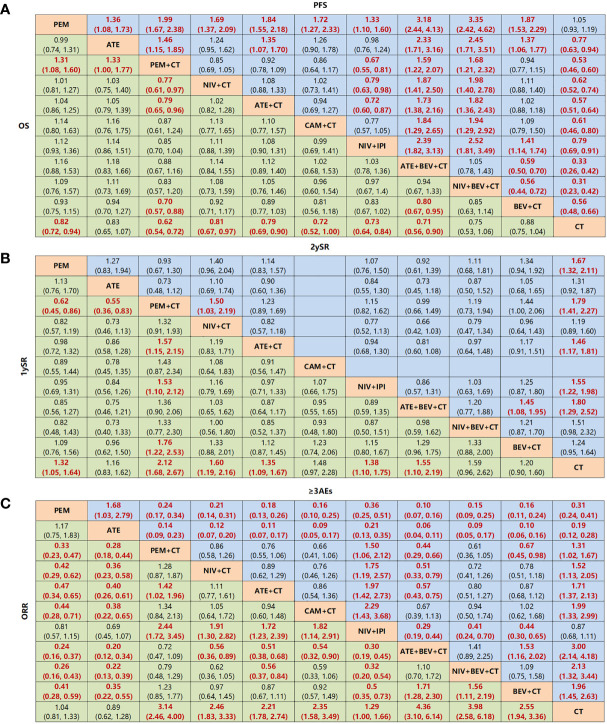
Network meta-analysis of specific immuno-related regimens with long-term survival in overall population. **(A)** Pooled HR (95% CrIs) for overall survival (OS) and progression-free survival (PFS) in comparisons of all treatment strategies. **(B)** Pooled OR (95% CrIs) for 1-year survival rate and 2-year survival rate in comparisons of all treatment strategies. Data in each cell are HR or (95% CrIs) for the comparison of row-defining treatment versus column-defining treatment. HR less than 1 and OR more than 1 favor upper-row treatment. Significant results are highlighted in red and bold. **(C)** Pooled OR (95% CrIs) for ORR and ≥3 AEs in comparisons of all treatment strategies. PEM, pembrolizumab; ATE, atezolizumab; NIV, nivolumab; CAM, camrelizumab; CEM, cemiplimab; BEV, bevacizumab; IPI, ipilimumab, CT, chemotherapy.

Among the treatments that resulted in an LS of 16.20 months or more for the all-comer population (**Figure 3**), PEM+CT (relative to CT: 0.62, 0.54~0.72) ranked first in mOS, followed by ATE+BEV+CT (0.71, 0.56~0.90), and the third is the NIV+IPI (0.73, 0.64~0.84) (**Figures 4A**, **5**, and **Supplementary Figure 3**). Almost all immuno-related therapies significantly prolonged OS compared with CT, and the OS of PEM+CT was prolonged significantly compared to NIV+CT (0.77, 0.61~0.97) and ATE+CT (0.79, 0.65~0.96) (**Figure 6A**). In terms of median progression-free survival (mPFS), the top three rankings of SUCRA cumulative probability were NIV+BEV+CT, ATE+BEV+CT, and PEM+CT(**Figure 5** and **Supplementary Figure 3**). Both ATE+BEV+CT and NIV+BEV+CT regimens were significantly superior to any other regimens in mPFS, and all regimens yielded greater benefits than CT alone. However, there was no significant difference between ATE+BEV+CT and NIV+BEV+CT in both OS and PFS (**Figure 6A**).

In terms of 1ySR, PEM+CT ranked first, followed by NIV+CT and ATE+BEV+CT according to SUCRA cumulative probability (**Figures 4B**, **5**, and **Supplementary Figure 3**). The 1ySR of PEM+CT was improved significantly compared to ATE+CT (OR 1.57, 1.15~2.15) and NIV+IPI (1.53, 1.10~2.12). Except for ATE, CAM+CT, and NIV+BEV+CT, almost all immuno-related treatments have significantly improved 1ySR compared to CT alone (**Figure 6B**). For 1yPR, PEM+CT, ATE+CT, ATE+BEV+CT, NIV+BEV+CT, and BEV+CT were significantly higher than that of CT (**Supplementary Figure 4**).

In terms of 2ySR, the top three cumulative probabilities of SUCRA were ATE+BEV+CT (versus CT: 1.80, 1.29~2.52), PEM+CT (1.79, 1.41~2.27), and PEM (1.67, 1.32~2.11). Moreover, 2ySR of NIV+IPI (1.55, 1.22~1.98) and ATE+CT (1.46, 1.17~1.81) also increased significantly in contrast to CT (**Figures 5**, **6B** and **Supplementary Figure 3**). It is worth noting that the 2ySR (1.45, 1.08~1.95) of ATE+BEV+CT was significantly higher compared with BEV+CT, although the 1ySR (1.29, 0.96~1.75) of ATE+BEV+CT was improved without significance (**Figure 6B**).

In terms of ORR, all regimens demonstrated significant benefits compared to CT. Of these, ATE+BEV+CT, NIV+BEV+CT, and PEM+CT ranked top three according to SUCRA (**Figure 5** and **Supplementary Figure 3**). ATE showed the lowest rates of ≥3 AEs among all regimens, and ≥3 AEs in ATE+BEV+CT were higher than those in any other treatments except CAM+CT and NIV+BEV+CT (**Figure 6C**).

### NMA for Subgroups

Different from the LS treatments in the ITT population, dual ICIs combined with CT (NIV+IPI+CT) were also included in the subgroup with PD-L1 ≥50%, in addition to single ICIs and ICIs combined with CT (PEM+CT, ATE+CT; **Supplementary Figures 5A–C**). All ICI-related regimens except DUR significantly improved OS compared to CT alone but showed no significant difference between them (**Supplementary Figure 6A**). In terms of SUCRA ranking and line chart ranking of mOS, CEM, PEM+CT, and ATE ranked top three (**Supplementary Figures 3**, **7**). In terms of 1ySR, almost all ICI-related treatments were significantly better than CT, the best of which was PEM+CT (versus CT: HR 2.95, 1.62~5.44), followed by CEM (2.25, 1.59~3.21) and NIV+IPI+CT (2.23, 1.21~4.18). For 2ySR, CEM (relative to CT: OR 2.75, 1.94~3.93), ATE (2.54, 1.40~4.71), and PEM (1.96, 1.49~2.57) ranked top three, and almost all ICI-related measures were significantly improved compared to CT (**Supplementary Figures 3** and **6B**).

In patients with high bTMB or tTMB (**Supplementary Figures 5D, E**), the treatment options that led to LS were PEM, NIV+IPI, PEM+CT, DUR+TRE, and DUR+TRE+CT. PEM (0.62, 0.48~0.80), NIV+IPI (0.68, 0.51~0.91), and PEM+CT (0.70, 0.52~0.96) ranked the top three in SUCRA cumulative probability rankings and line chart (**Supplementary Figures 3** and **6D**).

In the non-squamous subgroup, the regimens meeting the LS criteria included ICIs+CT (PEM+CT, ATE+CT, CAM+CT), dual ICIs (NIV+IPI), and ICIs combined with AA and CT (ATE+BEV+CT; **Supplementary Figures 5F, G**). For mOS, the SUCRA value of PEM+CT ranked the first, whose mOS was significantly improved compared to ATE+CT (0.71, 0.56~0.89), NIV+IPI (0.71, 0.55~0.91), and NIV+CT (0.65, 0.49~0.87), but without significant difference with ATE+BEV+CT (0.79, 0.58~1.08). ATE+BEV+CT and NIV+BEV+CT were superior to any other regimens in PFS, but no significant difference was found in OS and PFS between the two regimens (**Supplementary Figures 3** and **6E**).

Due to the limited data available of the non-highly selected population in the squamous subgroup, only PEM+CT and NIV+CT from KEYNOTE 407 (2) and CheckMate227-Part2 (7) studies were included. Both NIV+CT and PEM+CT significantly improved OS and PFS compared to CT in patients with squamous cell carcinoma (**Supplementary Figure 6F**).

### Consistency and Inconsistency Assessment

The density and trace diagram prove that our NMA results are stable and reliable (**Supplementary Figure 8**). According to DIC analysis, after choosing a random or fixed model, the difference between the DIC of the consistency and inconsistency models is within 5, and the consistency model is selected (**Supplementary Table 7**). Combining the direct comparison results of traditional frequency methods (**Supplementary Figure 9**) and the direct comparison between Bayesian models with NMA results (**Supplementary Figure 10**) and node analysis (**Supplementary Table 8**), there was no statistical difference between the direct and indirect comparisons in terms of the mOS and 1ySR and 2ySR.

### Sensitivity Analysis

In the integrated analysis, we excluded Lynch (28), Govindan (27), and MYSTIC (10) studies with the largest deviation in survival time to perform the sensitivity analysis. In the sensitivity analysis, the mOS of first-line immuno-related treatments was 17.32 (16.16, 18.47) months, and the 1ySR and 2ySR were 0.65 (0.62, 0.68) and 0.40 (0.35, 0.44), respectively (**Supplementary Table 6**). Furthermore, in order to ensure the reliability and robustness of the results, we conducted one sensitivity analysis by excluding phase II studies [KEYNOTE021 (19), Niho (15)] for NMA, and the treatments that ranked the best in the overall results remained unchanged (**Supplementary Figures 11, 12**). The equations should be inserted in editable format from the equation editor.

## Discussion

As mentioned above, the integrated analysis showed that POS for advanced NSCLC with immuno-related therapy was 16.20 months, with P1SR and P2SR of 63% and 37%. With 16.20 months as the standard of LS, the combination of ICIs and CT was more effective than either single or dual ICIs, and the addition of AA to ICI+CT resulted in the longest OS benefit and higher 1ySR and 2ySR. Considering the slow onset but longer duration of efficacy of immunotherapy, we are more inclined to regard the survival rate rather than median survival as the standard for evaluating the ICI-containing treatments, to reflect the efficacy character of immunotherapy more accurately. According to our pooled results, the POS was about 16~17 months, which was less than 1.5 years, while the P2SR was close to 40%, consistent with the recently published trend of the 5-year survival rate of 23.2% for first-line pembrolizumab treatment in advanced NSCLC (31). In this research, we focused on the 1ySR and 2ySR by integrated analysis, NMA, and ranking probability of various first-line ICI-containing treatments, to provide strong evidence for comprehensive comparison and assessment of the ability of each treatment to bring LS for advanced NSCLC in a relative longer observation time.

Immunotherapy combined with CT plays an important role in achieving LS in ITT patients with advanced wild-type NSCLC compared to either single or dual ICIs. The combination of ICIs and CT was superior to CT or ICIs alone (32), while there was no difference in mOS or survival rates between single ICI or dual ICIs without CT. The addition of CT helps not only overcome the shortcoming of the slow onset and the possible early ICI-related hyper-progression to improve the short-term response rate but also produce *in situ* vaccines to promote tumor antigen presentation to increase the efficacy of immunotherapy. Once activated, the immune response works continuously, making up for the short-effect duration of CT. Therefore, the synergism of immunotherapy and CT makes their combination the most reliable treatment strategy for LS of patients.

Among the top regimens, PEM+CT ranked the 1st, 1st, and 2nd in 1ySR, mOS, and 2ySR in the ITT population, respectively, while the corresponding rankings of ATE+BEV+CT were 4th, 2nd, and 1st. Notably, the pooled outcome of the ICIs+CT+AA strategy based on ATE+BEV+CT and NIV+BEV+CT has resulted in a P1SR of 74%, POS 19.5 months, and P2SR 49%, much better than other strategies. Therefore, adding AA on the basis of immuno-CT can not only yield a higher short-term effective rate and PFS but also translate them into a longer-term survival benefit. When considering the longest survival indicator 2ySR, the combination of ICIs+CT+AA was the strongest. As shown in **Figure 5**, if we arranged the SUCRA ranking values of efficacy indicators in a chronological order, only the immuno-AA-CT mode, instead of immuno-CT, dual immunotherapy, or AA-CT, showed a continuously rising tendency in the time span from 1 to 2 years of survival. The tendency may continue beyond 2 years. Consistently, another triplet similar to IMpower 150, NIV+BEV+CT failed to improve mOS and 1ySR compared to BEV+CT but significantly surpassed BEV+CT in 2ySR. It may be related to the synergistic role of ICIs and AA to target and transform the tumor microenvironment from immunosuppression into immune response, which takes a long time and functions for a long time. Obviously, further research is needed to determine which combination of immuno-AA-CT will lead to optimal survival. At the same time, we should pay full attention to the monitoring and handling of the side effects of the triplet therapy.

Another hot topic in clinical discussions is whether to apply single-ICI or ICI-combination therapy for patients with high PD-L1 expression. We found that among the regimens that resulted in LS in NSCLC with PD-L1 ≥50%, PEM+CT ranked first in 1ySR, while CEM monotherapy ranked first in mOS and 2ySR. Although immunotherapy shows a lasting and significant survival benefit over CT in the highly selected population, it is worth noting that dual ICIs or ICIs+CT failed to bring further benefits than single ICIs in terms of mOS, 1ySR, and 2ySR. Furthermore, the addition of CT to NIV+IPI also did not yield further benefits according to the survival indicators mOS, 1ySR, and 2ySR. It seems that the dominant position of PD-L1 inhibitors in patients with a high PD-L1 expression is unbreakable, possibly leaving limited space for CTLA-4 inhibitors or CT to further extend survival. Interestingly, the addition of AA improved 1ySR and final 2ySR in this population (although only IMpower150 data are available). However, we still need to identify and consider the small group of people with a high PD-L1 expression who fail to benefit from mono-ICI, especially those with hyper-progression, to give them individualized combination therapy such as a short course of CT at the beginning, to avoid rapid disease progression and poor prognosis.

To our knowledge, this is the first quantitative study to identify the optimal regimens for LS in patients with advanced wild-type NSCLC. By focusing on LS, we conducted NMA and SUCRA ranking of multidimensional survival indicators, such as mOS, 1ySR, and 2ySR, to comprehensively evaluate and identify the optimal treatments bringing LS for different populations with specific characteristics. Nevertheless, our study also has several limitations. First, several studies with moderate or high risk of bias are inevitably included, although most of them are phase 3 clinical trials. Second, the 1- or 2-year overall survival rate for which studies not directly presented were extracted through survival curve by software, especially the 2-year overall survival rate, might lead to some bias. Third, our idea for the definition of long-term survival is to simply define survival beyond the average overall OS level which is the result of the first integrated OS. Considering this method defining LS lacks the reference of statistical evidence, and we only use the integrated result as the standard to measure the pros and cons of various related treatment strategies, rather than promoting this definition as a rigorous statistical concept. In addition, the biomarkers associated with LS are only limited to high PD-L1 expression (33) or TMB (34) at present, so there are relatively few subgroup analyses. We found that the combination of immunotherapy with CT and AA is an effective mode to result in LS. The combined intervention targeting VEGF/VEGFR and immune checkpoints upon the immunosuppressive tumor microenvironment may robustly lead to superior effect than either of them, which may represent the future direction of treatments. Therefore, it is necessary to carry out research on the combination of various ICIs and AA therapies, with or without CT, in the treatment of advanced NSCLC, and strive to explore solutions to bring longer LS to these patients.

## Conclusions

In conclusion, the quantitative analysis of LS brought by immuno-related therapies to advanced wild-type NSCLC by integrated analysis and NMA will help expand the survival advantages of immunotherapy to the extreme and provide sufficient evidence for patients with different characteristics to choose individualized treatment regimens to obtain the maximum LS.

## Data Availability Statement

The raw data supporting the conclusions of this article will be made available by the authors, without undue reservation.

## Author Contributions

LL conceived and designed the study. XZ, QX, XY, MH, and XD independently assessed studies for possible inclusion and collected data. QX, XZ, LS, MH, SL, KH, JW, and XD analyzed the data. XZ and LL wrote the manuscript. LL is the guarantor of this study and accepts full responsibility for the work, had access to the data, and controlled the decision to publish. The corresponding authors attest that all listed authors meet the authorship criteria and that no other person meeting the criteria has been omitted. All authors contributed to the article and approved the submitted version.

## Conflict of Interest

The authors declare that the research was conducted in the absence of any commercial or financial relationships that could be construed as a potential conflict of interest.

## Publisher’s Note

All claims expressed in this article are solely those of the authors and do not necessarily represent those of their affiliated organizations, or those of the publisher, the editors and the reviewers. Any product that may be evaluated in this article, or claim that may be made by its manufacturer, is not guaranteed or endorsed by the publisher.
