# Supplementary material for: What Is Long-Term Survival and Which First-Line Immunotherapy Brings Long-Term Survival for Advanced Wild-Type Non-Small Cell Lung Cancer: A Network Meta-Analysis Based on Integrated Analysis
Source: Front Immunol. 2022 Apr 5;13:764643. doi: 10.3389/fimmu.2022.764643 (PMC9016897; doi:10.3389/fimmu.2022.764643)
Supplement: Supplementary file 1 [file DataSheet_1.pdf]

Supplementary Material

1 Supplementary Figures

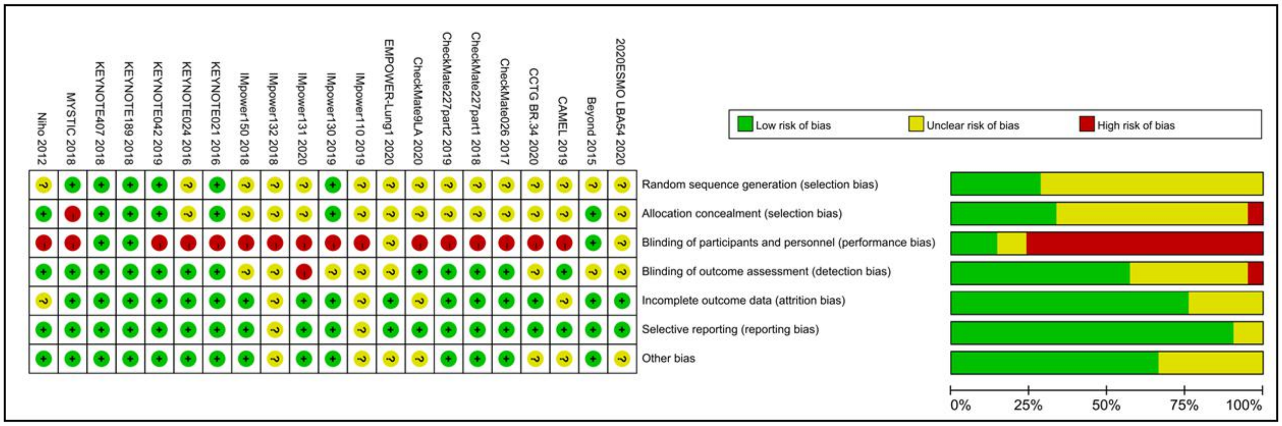

Supplementary Figure 1. Summary of results from bias risk assessment of studies using the Cochrane risk of bias tool.

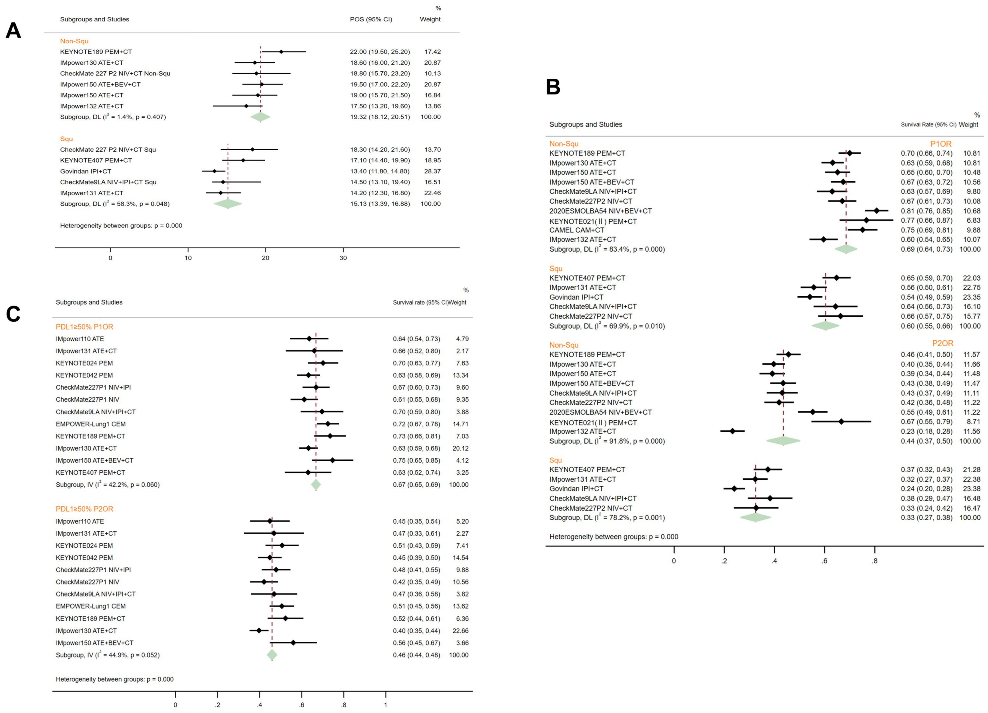

Supplementary Figure 2. Pooled survival outcomes from integrated analysis for subgroups of the median overall survival (OS) time, 1-year OS rate and 2-year OS rate of different therapy strategies containing Immune checkpoint inhibitors in patients with advanced wild-type NSCLC. (A) Median OS of non-squamous and squamous subgroups. (B) 1-year OS rate and 2-year OS rate of non-squamous and squamous subgroups. (C) 1-year OS rate and 2-year OS rate of PD-L1≥50% (or TC/IC=3) subgroups. PD-L1: programmed-death ligand 1; TC: tumor cells; IC: tumor-infiltrating immune cells; Squ: squamous; Non-squ: non-squamous; POS: pooled median overall survival; P1OR: pooled 1-year OS rate; P2OR: pooled 2-year OS rate

A

	Overall population					Non-squ	Squ	PD-L1≥50% TC/IC=3			High-TMB
Treatment	OS	ORR	≥3AEs	1yOR	2yOR	OS	OS	OS	1yOR	2yOR	OS
PEM+CT	**0.933**	0.787	0.434	**0.961**	0.829	**0.970**	0.721	0.722	**0.905**	0.459	0.599
ATE+BEV+CT	0.724	**0.958**	**0.986**	0.669	**0.834**	0.712	NA	NA	NA	NA	NA
NIV+IPI	0.678	0.282	0.215	0.500	0.618	0.499	NA	0.467	0.501	0.455	0.644
CAM+CT	0.657	0.555	0.756	0.582	NA	0.646	NA	NA	NA	NA	NA
NIV+BEV+CT	0.582	0.908	0.798	0.669	0.572	0.579	NA	NA	NA	NA	NA
ATE+CT	0.488	0.484	0.627	0.452	0.512	0.504	NA	0.585	0.289	0.429	NA
NIV+CT	0.429	0.587	0.543	0.702	0.239	0.325	0.771	NA	NA	NA	NA
PEM	0.392	0.150	0.098	0.415	0.730	NA	NA	0.545	0.549	0.637	**0.769**
ATE	0.379	0.055	0.002	0.248	0.372	NA	NA	0.705	0.514	0.820	0.494
BEV+CT	0.215	0.621	0.752	0.263	0.264	0.234	NA	NA	NA	NA	NA
CT	0.024	0.114	0.289	0.039	0.031	0.031	0.007	0.012	0.015	0.023	0.160
CEM	NA	NA	NA	NA	NA	NA	NA	**0.786**	0.766	**0.909**	NA
NIV+IPI+CT	NA	NA	NA	NA	NA	NA	NA	0.562	0.720	0.552	NA
DUR	NA	NA	NA	NA	NA	NA	NA	0.344	NA	NA	0.602
NIV	NA	NA	NA	NA	NA	NA	NA	0.271	0.240	0.216	0.152
DUR+TRE	NA	NA	NA	NA	NA	NA	NA	NA	NA	NA	0.568
DUR+TRE+CT	NA	NA	NA	NA	NA	NA	NA	NA	NA	NA	0.511

	Overall population				Non-squ	Squ	PD-L1≥50% TC/IC=3		High-TMB
Treatment	PFS	ORR	≥3AEs	1yPR	PFS	PFS	PFS	1yPR	PFS
NIV+BEV+CT	**0.963**	0.908	0.798	**0.924**	**0.947**	NA	NA	NA	NA
ATE+BEV+CT	0.937	**0.958**	**0.986**	0.886	0.909	NA	NA	NA	NA
PEM+CT	0.731	0.787	0.434	0.497	0.666	0.640	**0.833**	0.650	**0.981**
BEV+CT	0.637	0.621	0.752	0.740	0.481	NA	NA	NA	NA
ATE+CT	0.601	0.484	0.627	0.604	0.437	NA	0.706	0.494	NA
CAM+CT	0.521	0.555	0.756	0.318	0.349	NA	NA	NA	NA
NIV+CT	0.488	0.587	0.543	0.419	0.211	0.860	NA	NA	NA
ATE	0.273	0.055	0.002	0.425	NA	NA	0.476	0.380	0.681
NIV+IPI	0.249	0.282	0.215	0.438	NA	NA	0.493	0.613	0.683
CT	0.079	0.114	0.289	0.093	0.000	0.000	0.112	0.087	0.157
PEM	0.022	0.150	0.098	0.158	NA	NA	0.448	0.437	NA
CEM	NA	NA	NA	NA	NA	NA	0.587	**0.847**	NA
NIV	NA	NA	NA	NA	NA	NA	0.346	0.491	0.618
DUR+TRE+CT	NA	NA	NA	NA	NA	NA	NA	NA	0.350
DUR	NA	NA	NA	NA	NA	NA	NA	NA	0.326
DUR+TRE	NA	NA	NA	NA	NA	NA	NA	NA	0.205

B

	Overall population					Non-squ	Squ	PD-L1≥50% TC/IC=3			High-TMB
Treatment	OS	ORR	≥3AEs	1yOR	2yOR	OS	OS	OS	1yOR	2yOR	OS
PEM+CT	**1**	3	7	**1**	2	**1**	2	2	**1**	5	4
ATE+BEV+CT	2	**1**	**1**	4	**1**	2	NA	NA	NA	NA	NA
NIV+IPI	3	8	9	6	4	6	NA	7	6	6	2
CAM+CT	4	6	3	5	NA	3	NA	NA	NA	NA	NA
NIV+BEV+CT	5	2	2	3	5	4	NA	NA	NA	NA	NA
ATE+CT	6	7	5	7	6	5	NA	4	7	7	NA
NIV+CT	7	5	6	2	9	7	**1**	NA	NA	NA	NA
PEM	8	9	10	8	3	NA	NA	6	4	3	**1**
ATE	9	11	11	10	7	NA	NA	3	5	2	7
BEV+CT	10	4	4	9	8	8	NA	NA	NA	NA	NA
CT	11	10	8	11	10	9	3	10	9	9	8
CEM	NA	NA	NA	NA	NA	NA	NA	**1**	2	**1**	NA
NIV+IPI+CT	NA	NA	NA	NA	NA	NA	NA	5	3	4	NA
DUR	NA	NA	NA	NA	NA	NA	NA	8	NA	NA	3
NIV	NA	NA	NA	NA	NA	NA	NA	9	8	8	9
DUR+TRE	NA	NA	NA	NA	NA	NA	NA	NA	NA	NA	5
DUR+TRE+CT	NA	NA	NA	NA	NA	NA	NA	NA	NA	NA	6

	Overall population				Non-squ	Squ	PD-L1≥50% TC/IC=3		High-TMB
Treatment	PFS	ORR	≥3AEs	1yPR	PFS	PFS	PFS	1yPR	PFS
NIV+BEV+CT	**1**	2	2	**1**	**1**	NA	NA	NA	NA
ATE+BEV+CT	2	**1**	**1**	2	2	NA	NA	NA	NA
PEM+CT	3	3	7	5	3	2	**1**	2	**1**
BEV+CT	4	4	4	3	4	NA	NA	NA	NA
ATE+CT	5	7	5	4	5	NA	2	4	NA
CAM+CT	6	6	3	9	6	NA	NA	NA	NA
NIV+CT	7	5	6	8	7	**1**	NA	NA	NA
ATE	8	11	11	7	NA	NA	5	7	3
NIV+IPI	9	8	9	6	NA	NA	4	3	2
CT	10	10	8	11	8	3	8	8	8
PEM	11	9	10	10	NA	NA	6	6	NA
CEM	NA	NA	NA	NA	NA	NA	3	**1**	NA
NIV	NA	NA	NA	NA	NA	NA	7	5	4
DUR+TRE+CT	NA	NA	NA	NA	NA	NA	NA	NA	5
DUR	NA	NA	NA	NA	NA	NA	NA	NA	6
DUR+TRE	NA	NA	NA	NA	NA	NA	NA	NA	7

Supplementary Figure 3. Bayesian ranking profiles of comparable treatments with long-term survival time on efficacy and safety for patients with advanced wild-type NSCLC. (A) Percentage of the surface under the cumulative ranking area (SUCRA) in each cell indicates the probability of each treatment being ranked from first (high value) to last (low value) on survival indicators for overall population and subgroups. (B) Number in each cell indicates the probability of the ranking of each treatment on survival indicators for overall population and subgroups. PEM: pembrolizumab; ATE: atezolizumab; NIV: nivolumab; DUR: durvalumab; TRE: tremelimumab; IPI: ipilimumab; CAM: camrelizumab; CEM: cemiplimab; BEV: bevacizumab; CT: chemotherapy; OS: overall survival; PFS: progression-free survival; ORR: objective response rate; ⩾3AEs: grade ⩾3 adverse events; 1yOR: 1-year OS rate; 2yOR: 2-year OS rate; 1yPR: 1-year PFS rate; Squ: squamous; Non-squ: nonsquamous; PD-L1: programmed-death ligand 1; TMB: tumor mutation burden； TC: tumor cells; IC: tumor-infiltrating immune cells

1yPR

**PEM**										
0.48 (0.06, 3.89)	**ATE**									
0.41 (0.08, 2.21)	0.84 (0.16, 4.67)	**PEM+CT**								
0.49 (0.06, 3.72)	1.01 (0.13, 7.85)	1.20 (0.22, 6.32)	**NIV+CT**							
0.32 (0.05, 1.68)	0.66 (0.11, 3.50)	0.78 (0.21, 2.58)	0.65 (0.11, 3.38)	**ATE+CT**						
0.63 (0.08, 5.04)	1.31 (0.16, 10.4)	1.55 (0.28, 8.41)	1.29 (0.16, 10.05)	1.98 (0.37, 11.91)	**CAM+CT**					
0.46 (0.06, 3.68)	0.97 (0.12, 7.75)	1.15 (0.21, 6.12)	0.96 (0.13, 7.40)	1.46 (0.28, 8.65)	0.74 (0.09, 5.84)	**NIV+IPI**				
0.12 (0.01, 0.87)	0.26 (0.03, 1.84)	0.30 (0.05, 1.49)	0.25 (0.03, 1.77)	0.39 (0.09, 1.46)	0.20 (0.02, 1.41)	0.27 (0.03, 1.86)	**ATE+BEV+CT**			
0.10 (0.01, 0.79)	0.20 (0.02, 1.69)	0.24 (0.03, 1.40)	0.20 (0.02, 1.62)	0.31 (0.04, 1.68)	0.15 (0.01, 1.29)	0.21 (0.02, 1.69)	0.79 (0.10, 5.30)	**NIV+BEV+CT**		
0.23 (0.03, 1.14)	0.47 (0.07, 2.35)	0.55 (0.12, 1.76)	0.46 (0.07, 2.26)	0.71 (0.21, 1.87)	0.36 (0.05, 1.81)	0.49 (0.07, 2.35)	1.84 (0.44, 6.63)	2.33 (0.55, 9.86)	**BEV+CT**	
1.08 (0.25, 4.69)	2.24 (0.51, 9.71)	**2.65 (1.11, 6.17)**	2.21 (0.53, 9.36)	**3.40 (1.44, 9.01)**	1.72 (0.39, 7.46)	2.32 (0.54, 9.74)	**8.72 (2.23, 42.81)**	**11.11 (2.25, 78.06)**	**4.81 (2.05, 15.60)**	**CT**

Supplementary Figure 4. Network meta-analysis of specific immuno-related regimens with long-term survival on 1-year progression-free survival rate in overall population.

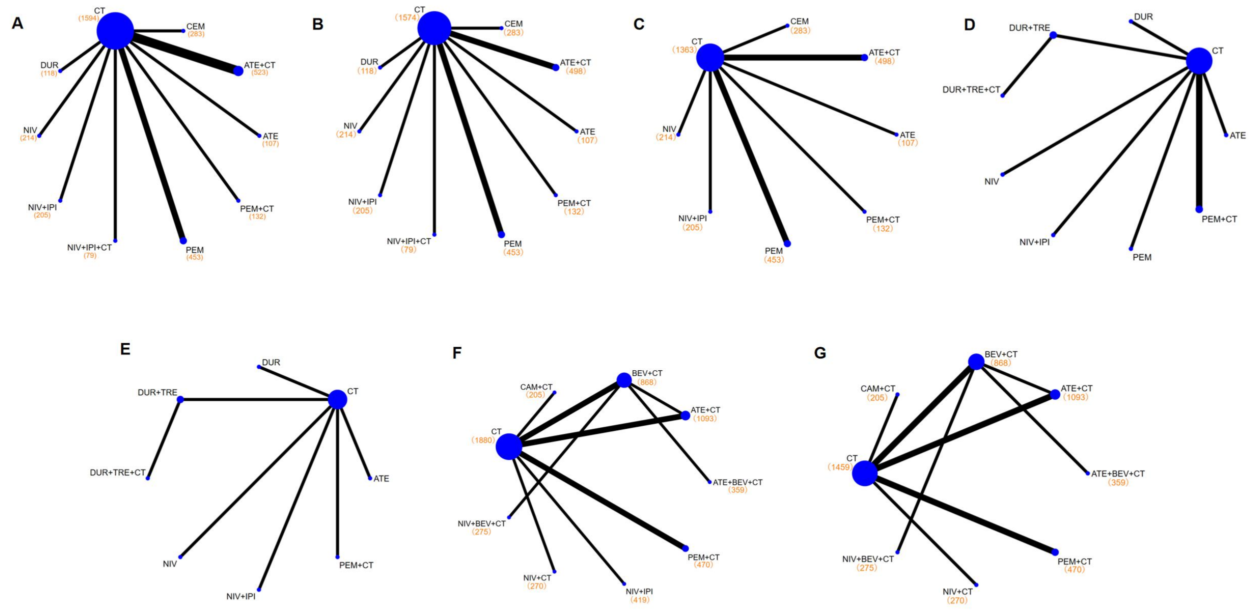

Supplementary Figure 5. Network diagrams of comparisons on different outcomes of treatments with long-term survival time in subgroups analyses. (A) comparisons on overall survival (OS) in patients with PD-L1⩾50% (or TC/IC=3) subgroup. (B) comparisons on 1-year OS rate and 2-year OS rate in patients with PD-L1⩾50% (or TC/IC=3) subgroup. (C) comparisons on progression-free survival (PFS) and 1-year PFS rate in patients with PD-L1⩾50% (or TC/IC=3) subgroup. (D) comparisons on OS of high blood tumor mutation burden (bTMB) or tissue tumor mutation burden (tTMB) subgroup. (E) comparisons on PFS of high bTMB or tTMB subgroup. (F) comparisons on OS of non-squamous subgroup. (G) comparisons on PFS of non-squamous subgroup. Each circular node represents a type of treatment. Each line represents a type of head-to-head comparison. The size of the nodes and the thickness of the lines are weighted according to the number of studies evaluating each treatment and direct comparison, respectively. The total number of patients receiving a treatment was shown in brackets. PEM: pembrolizumab; ATE: atezolizumab; NIV: nivolumab; CAM: camrelizumab; DUR: durvalumab; TRE: tremelimumab; IPI: ipilimumab; BEV: bevacizumab; CEM: cemiplimab; CT: chemotherapy; PD-L1: programmed-death ligand 1; TC: tumor cells; IC: tumor-infiltrating immune cells

A (upper triangle: PFS; lower triangle: OS)

**PEM**	0.87 (0.22, 3.41)	1.04 (0.25, 4.10)	1.21 (0.30, 4.74)		1.87 (0.46, 7.41)	1.42 (0.45, 4.45)	1.06 (0.26, 4.11)		0.65 (0.29, 1.43)
0.85 (0.64, 1.12)	**NIV**	1.19 (0.24, 5.93)	1.39 (0.28, 6.81)		2.14 (0.43, 10.56)	1.63 (0.40, 6.61)	1.21 (0.39, 3.79)		0.75 (0.24, 2.31)
1.12 (0.73, 1.73)	1.33 (0.83, 2.11)	**ATE**	1.17 (0.24, 5.82)		1.80 (0.36, 9.13)	1.37 (0.33, 5.67)	1.02 (0.20, 5.09)		0.63 (0.20, 2.00)
1.18 (0.84, 1.65)	1.39 (0.94, 2.03)	1.04 (0.63, 1.73)	**CEM**		1.54 (0.31, 7.65)	1.17 (0.29, 4.77)	0.87 (0.18, 4.26)		0.54 (0.18, 1.66)
0.88 (0.62, 1.26)	1.04 (0.70, 1.55)	0.78 (0.47, 1.31)	0.75 (0.48, 1.16)	**DUR**					
1.14 (0.75, 1.72)	1.34 (0.86, 2.10)	1.01 (0.58, 1.76)	0.97 (0.59, 1.57)	1.29 (0.78, 2.12)	**PEM+CT**	0.76 (0.19, 3.14)	0.56 (0.11, 2.81)		0.35 (0.11, 1.10)
1.03 (0.71, 1.48)	1.21 (0.81, 1.82)	0.91 (0.54, 1.54)	0.87 (0.56, 1.37)	1.17 (0.74, 1.85)	0.90 (0.55, 1.50)	**ATE+CT**	0.74 (0.18, 2.99)		0.46 (0.20, 1.06)
0.96 (0.71, 1.28)	1.13 (0.80, 1.59)	0.85 (0.53, 1.36)	0.81 (0.55, 1.21)	1.09 (0.73, 1.63)	0.84 (0.54, 1.33)	0.93 (0.62, 1.41)	**NIV+IPI**		0.62 (0.20, 1.91)
1.01 (0.66, 1.57)	1.20 (0.75, 1.91)	0.90 (0.51, 1.60)	0.86 (0.52, 1.44)	1.15 (0.69, 1.93)	0.89 (0.51, 1.56)	0.99 (0.59, 1.67)	1.06 (0.66, 1.71)	**NIV+IPI+CT**	
**0.67 (0.57, 0.78)**	**0.79 (0.62, 1.00)**	**0.60 (0.40, 0.89)**	**0.57 (0.42, 0.77)**	0.76 (0.55, 1.05)	**0.59 (0.40, 0.86)**	**0.65 (0.47, 0.91)**	**0.70 (0.55, 0.90)**	**0.66 (0.44, 0.99)**	**CT**

B (upper triangle: 2yOR; lower triangle: 1yOR)

**PEM**	1.51 (0.93, 2.45)	0.77 (0.39, 1.49)	0.71 (0.46, 1.11)	1.19 (0.62, 2.27)	1.21 (0.80, 1.84)	1.20 (0.74, 1.94)	1.07 (0.55, 2.08)	**1.96 (1.49, 2.57)**
1.35 (0.83, 2.17)	**NIV**	0.51 (0.24, 1.05)	0.47 (0.28, 0.81)	0.79 (0.38, 1.59)	0.8 (0.48, 1.34)	0.79 (0.54, 1.16)	0.71 (0.34, 1.47)	1.30 (0.87, 1.94)
1.03 (0.55, 1.92)	0.76 (0.38, 1.52)	**ATE**	0.92 (0.46, 1.87)	1.54 (0.66, 3.61)	1.57 (0.80, 3.12)	1.55 (0.75, 3.24)	1.39 (0.59, 3.29)	**2.54 (1.40, 4.71)**
0.80 (0.52, 1.24)	0.60 (0.35, 1.01)	0.78 (0.40, 1.51)	**CEM**	1.67 (0.83, 3.30)	**1.70 (1.05, 2.73)**	1.68 (0.99, 2.89)	1.50 (0.75, 3.02)	**2.75 (1.94, 3.93)**
0.61 (0.31, 1.17)	**0.45 (0.22, 0.93)**	0.59 (0.26, 1.34)	0.76 (0.38, 1.53)	**PEM+CT**	1.02 (0.52, 2.00)	1.01 (0.49, 2.08)	0.90 (0.39, 2.12)	1.65 (0.92, 3.01)
1.27 (0.85, 1.91)	0.95 (0.58, 1.55)	1.24 (0.66, 2.33)	**1.59 (1.00, 2.54)**	**2.10 (1.06, 4.16)**	**ATE+CT**	0.99 (0.59, 1.66)	0.89 (0.45, 1.76)	**1.62 (1.18, 2.24)**
1.06 (0.65, 1.71)	0.78 (0.52, 1.17)	1.03 (0.51, 2.05)	1.32 (0.77, 2.26)	1.74 (0.83, 3.60)	0.83 (0.50, 1.37)	**NIV+IPI**	0.9 (0.43, 1.84)	**1.63 (1.09, 2.45)**
0.81 (0.41, 1.58)	0.60 (0.29, 1.25)	0.79 (0.34, 1.81)	1.01 (0.49, 2.05)	1.33 (0.55, 3.14)	0.63 (0.32, 1.25)	0.77 (0.36, 1.60)	**NIV+IPI+CT**	**1.83 (1.00, 3.36)**
**1.80 (1.38, 2.35)**	1.34 (0.90, 1.99)	**1.75 (1.00, 3.09)**	**2.25 (1.59, 3.21)**	**2.95 (1.62, 5.44)**	**1.41 (1.05, 1.91)**	**1.71 (1.14, 2.58)**	**2.23 (1.21, 4.18)**	**CT**

C (1yPR)

**PEM**							
0.86 (0.06, 13.61)	**NIV**						
1.18 (0.08, 19.85)	1.39 (0.06, 33.15)	**ATE**					
0.28 (0.02, 4.18)	0.32 (0.01, 7.41)	0.23 (0.01, 5.51)	**CEM**				
0.55 (0.04, 8.99)	0.65 (0.03, 15.30)	0.47 (0.02, 11.10)	2.01 (0.09, 48.12)	**PEM+CT**			
0.88 (0.09, 8.34)	1.02 (0.06, 15.43)	0.74 (0.04, 11.13)	3.15 (0.20, 47.07)	1.58 (0.09, 24.44)	**ATE+CT**		
0.64 (0.04, 10.18)	0.75 (0.08, 6.91)	0.54 (0.02, 13.33)	2.31 (0.10, 52.86)	1.15 (0.05, 28.17)	0.73 (0.05, 12.05)	**NIV+IPI**	
2.50 (0.55, 12.48)	2.94 (0.31, 27.04)	2.12 (0.22, 19.94)	9.10 (0.98, 83.3)	4.52 (0.47, 43.36)	2.88 (0.59, 14.93)	3.94 (0.43, 35.44)	**CT**

D (upper triangle: PFS; lower triangle: OS)

**PEM**								
0.56 (0.31, 1.02)	**NIV**	1.11 (0.50, 2.45)	0.72 (0.37, 1.39)	**1.94 (1.00, 3.74)**	1.07 (0.59, 1.93)	0.64 (0.33, 1.22)	0.73 (0.30, 1.74)	0.62 (0.38, 1.01)
0.80 (0.36, 1.79)	1.42 (0.56, 3.61)	**ATE**	0.65 (0.30, 1.41)	1.75 (0.81, 3.79)	0.97 (0.47, 1.98)	0.58 (0.27, 1.24)	0.66 (0.25, 1.72)	0.56 (0.30, 1.05)
0.89 (0.55, 1.43)	1.57 (0.80, 3.08)	1.10 (0.47, 2.59)	**DUR**	**2.69 (1.44, 5.02)**	1.48 (0.85, 2.59)	0.89 (0.48, 1.64)	1.01 (0.43, 2.35)	0.86 (0.55, 1.34)
0.88 (0.59, 1.32)	1.56 (0.84, 2.90)	1.10 (0.48, 2.48)	1.00 (0.60, 1.66)	**PEM+CT**	**0.55 (0.32, 0.97)**	**0.33 (0.18, 0.61)**	**0.37 (0.16, 0.88)**	**0.32 (0.21, 0.50)**
0.91 (0.62, 1.34)	1.62 (0.88, 2.98)	1.13 (0.50, 2.55)	1.03 (0.63, 1.70)	1.03 (0.68, 1.58)	**NIV+IPI**	0.60 (0.35, 1.04)	0.68 (0.31, 1.51)	**0.58 (0.41, 0.81)**
0.86 (0.53, 1.39)	1.53 (0.78, 3.00)	1.07 (0.45, 2.53)	0.97 (0.55, 1.73)	0.98 (0.59, 1.63)	0.94 (0.57, 1.56)	**DUR+TRE**	1.14 (0.64, 2.04)	0.97 (0.63, 1.49)
0.82 (0.31, 2.19)	1.46 (0.49, 4.32)	1.02 (0.30, 3.42)	0.93 (0.33, 2.59)	0.93 (0.34, 2.51)	0.90 (0.33, 2.41)	0.95 (0.41, 2.24)	**DUR+TRE+CT**	0.85 (0.41, 1.76)
**0.62 (0.48, 0.80)**	1.10 (0.64, 1.88)	0.77 (0.36, 1.64)	0.70 (0.47, 1.05)	**0.70 (0.52, 0.96)**	**0.68 (0.51, 0.91)**	0.72 (0.48, 1.08)	0.76 (0.29, 1.94)	**CT**

E (upper triangle: PFS; lower triangle: OS)

**PEM+CT**	**0.74 (0.57, 0.96)**	0.87 (0.70, 1.06)	0.81 (0.59, 1.12)		**1.50 (1.12, 2.00)**	**1.58 (1.12, 2.22)**	0.88 (0.70, 1.11)	**0.49 (0.42, 0.59)**
**0.65 (0.49, 0.87)**	**NIV+CT**	1.17 (0.93, 1.48)	1.10 (0.78, 1.55)		**2.03 (1.49, 2.75)**	**2.14 (1.49, 3.05)**	1.20 (0.93, 1.54)	**0.67 (0.55, 0.82)**
**0.71 (0.56, 0.89)**	1.09 (0.84, 1.41)	**ATE+CT**	0.94 (0.69, 1.26)		**1.73 (1.38, 2.16)**	**1.82 (1.36, 2.43)**	1.02 (0.88, 1.18)	**0.57 (0.51, 0.64)**
0.78 (0.53, 1.14)	1.19 (0.80, 1.78)	1.10 (0.77, 1.57)	**CAM+CT**		**1.85 (1.29, 2.65)**	**1.94 (1.30, 2.91)**	1.09 (0.79, 1.50)	**0.61 (0.46, 0.80)**
**0.71 (0.55, 0.91)**	1.09 (0.82, 1.44)	1.00 (0.81, 1.24)	0.91 (0.63, 1.32)	**NIV+IPI**				
0.79 (0.58, 1.08)	1.21 (0.87, 1.69)	1.12 (0.89, 1.40)	1.02 (0.67, 1.53)	1.12 (0.83, 1.49)	**ATE+BEV+CT**	1.05 (0.78, 1.43)	**0.59 (0.50, 0.70)**	**0.33 (0.26, 0.42)**
0.74 (0.51, 1.10)	1.14 (0.76, 1.72)	1.05 (0.76, 1.46)	0.96 (0.60, 1.54)	1.05 (0.72, 1.53)	0.94 (0.67, 1.33)	**NIV+BEV+CT**	**0.56 (0.44, 0.72)**	**0.31 (0.23, 0.42)**
**0.63 (0.49, 0.82)**	0.97 (0.74, 1.28)	0.89 (0.77, 1.03)	0.81 (0.56, 1.18)	0.89 (0.71, 1.13)	**0.80 (0.67, 0.95)**	0.85 (0.63, 1.14)	**BEV+CT**	**0.56 (0.48, 0.66)**
**0.56 (0.46, 0.68)**	0.86 (0.69, 1.07)	**0.79 (0.69, 0.90)**	**0.72 (0.52, 1.00)**	**0.79 (0.67, 0.93)**	**0.71 (0.56, 0.90)**	0.75 (0.54, 1.05)	0.88 (0.75, 1.05)	**CT**

F (upper triangle: PFS; lower triangle: OS)

**PEM+CT**	1.12 (0.77, 1.62)	**0.57 (0.47, 0.69)**
1.03 (0.70, 1.52)	**NIV+CT**	**0.51 (0.37, 0.70)**
**0.71 (0.58, 0.87)**	**0.69 (0.50, 0.96)**	**CT**

Supplementary Figure 6. Network meta-analysis of specific immuno-related regimens with long-term survival in subgroups analyses. (A) Pooled HR (95% CrIs) for overall survival (OS) and progression-free survival (PFS) in patients with PD-L1⩾50% (or TC/IC=3) subgroup. (B) Pooled OR (95% CrIs) for 1-year OS rate and 2-year OS rate in patients with PD-L1⩾50% (or TC/IC=3) subgroup. (C) Pooled OR (95% CrIs) for 1-year PFS rate in patients with PD- L1⩾50% (or TC/IC=3) subgroup. (D) Pooled HR (95% CrIs) for OS and PFS of high blood tumor mutation burden (bTMB) or tissue tumor mutation burden (tTMB) subgroup. (E) Pooled HR (95% CrIs) for OS and PFS of non-squamous subgroup. (F) Pooled HR (95% CrIs) for OS and PFS of squamous subgroup. Data in each cell are HR or OR (95% CrIs) for the comparison of row-defining treatment versus column-defining treatment. HR less than 1 and OR more than 1 favour upper-row treatment. Significant results are highlighted in red and bold. PEM: pembrolizumab; ATE: atezolizumab; NIV: nivolumab; DUR: durvalumab; TRE: tremelimumab; IPI: ipilimumab; CAM: camrelizumab; CEM: cemiplimab; BEV: bevacizumab; CT: chemotherapy; 1yOR: 1-year OS rate; 2yOR: 2-year OS rate; 1yPR: 1-year PFS rate; PD-L1: programmed-death ligand 1; TC: tumor cells; IC: tumor-infiltrating immune cells

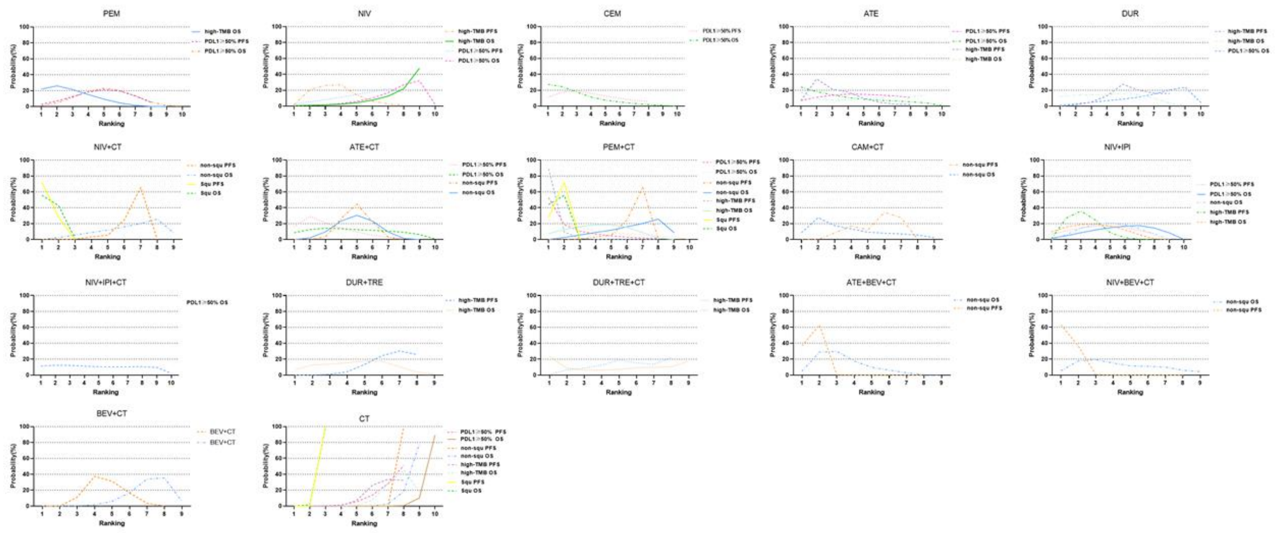

Supplementary Figure 7. Bayesian ranking profiles indicating the probability of each comparable treatment of subgroups with long-term survival time for patients with advanced NSCLC. OS: overall survival; PFS: progression-free survival; ORR: objective response rate; Non-squ: nonsquamous subgroup; Squ: squamous subgroup; PD-L1: programmed- death ligand 1; TC: tumor cells; IC: tumor-infiltrating immune cells. PEM:pembrolizumab; ATE:atezolizumab; NIV:nivolumab; DUR:durvalumab; TRE:tremelimumab; IPI:ipilimumab; CAM:camrelizumab; CEM:cemiplimab; BEV:bevacizumab; CT:chemotherapy

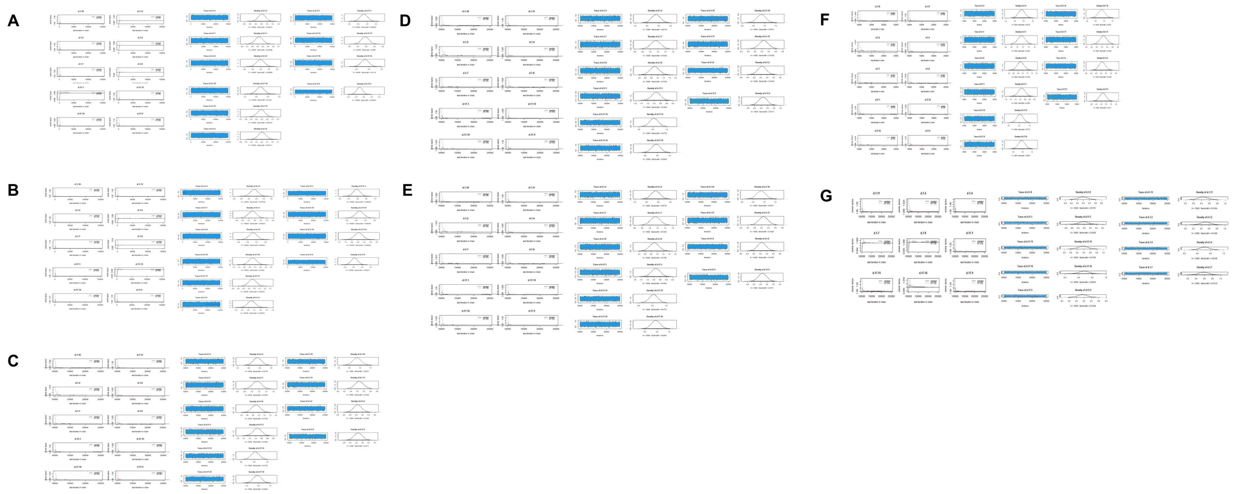

Supplementary Figure 8. Convergence of the four chains established by inspection of the Brooks-Gelman-Rubin diagnostic and the trace and density plot of overall survival (OS) (A), progression-free survival (PFS) (B), objective response rate (C), grade ⩾ 3 adverse events (D), 1-year OS rate (E),1-year PFS rate (F) , 2-year OS rate (G).

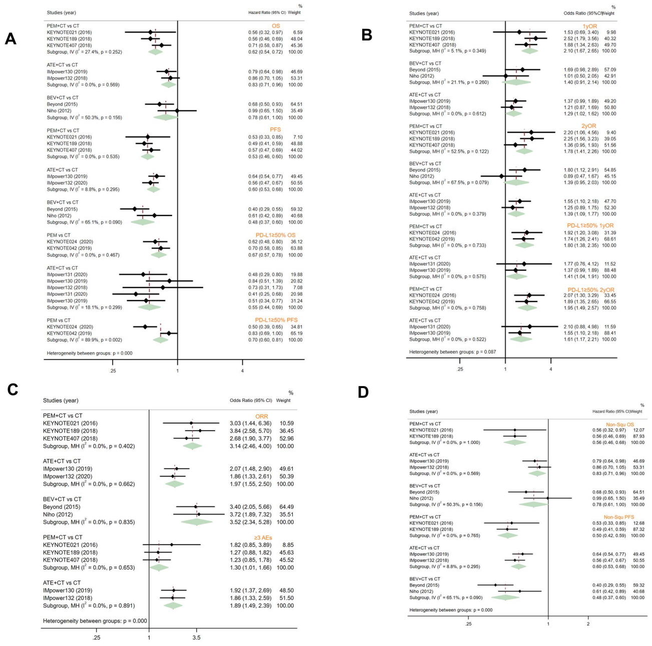

Supplementary Figure 9. Forest plots depicting results of head-to-head comparisons according to frequentist pairwise meta-analyses of specific immuno-related regimens with long-term survival on different outcomes in advanced wild-type NSCLC. (A) Outcomes for overall survival (OS) and progression-free survival (PFS) in overall population and PD-L1⩾50% (or TC/IC=3) subgroup. (B) Outcomes for 1-year OS rate and 2-year OS rate in overall population and in PD-L1⩾50% (or TC/IC=3) subgroup. (C) Outcomes for objective response rate (ORR) and adverse events of grade 3 or higher (⩾ 3 AEs) in overall population. (D) Outcomes for OS and PFS in non-squamous subgroups. PEM: pembrolizumab; ATE: atezolizumab; BEV: bevacizumab; CT: chemotherapy; PD-L1: programmed-death ligand 1; TC: tumor cells; IC: tumor-infiltrating immune cells; 1yOR: 1-year OS rate; 2yOR: 2-year OS rate

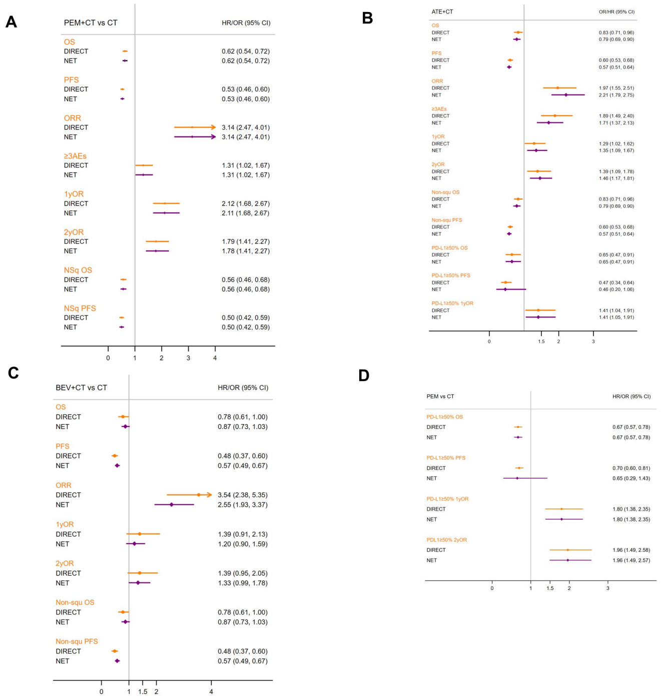

Supplementary Figure 10. Forest plots depicting results of head-to-head comparisons according to Bayesian pairwise and network meta-analysis of specific immuno-related regimens with long-term survival. Results of all comparisons in overall population, non-squamous and PD-L1⩾50% (or TC/IC=3) subgroups were consistent between pairwise and network meta-analysis. PEM: pembrolizumab; ATE: atezolizumab; BEV: bevacizumab; CT: chemotherapy; TC: tumor cells; IC: tumor-infiltrating immune cells; OS: overall survival; PFS: progression- free survival; ORR: objective response rate; 1yOR: 1-year OS rate; 2yOR: 2-year OS rate; Non-squ: nonsquamous; PD-L1: programmed-death ligand 1; TC: tumor cells; IC: tumor-infiltrating immune cells

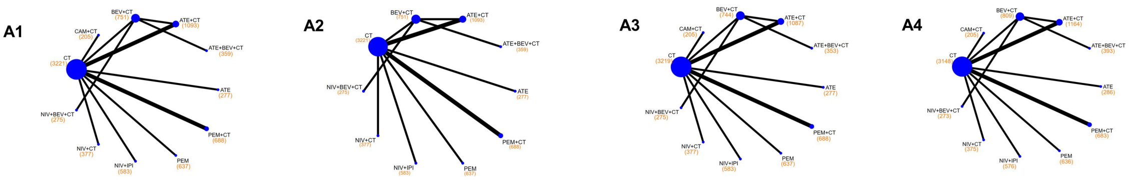

B

PFS (upper triangle) / OS (lower triangle)

**PEM**	**1.36 (1.08, 1.72)**	**1.99 (1.66, 2.39)**	**1.69 (1.37, 2.09)**	**1.85 (1.56, 2.19)**	**1.72 (1.27, 2.33)**	**1.33 (1.10, 1.60)**	**3.24 (2.46, 4.25)**	**3.41 (2.45, 4.74)**	**1.91 (1.54, 2.37)**	1.05 (0.93, 1.19)
0.99 (0.74, 1.31)	**ATE**	**1.46 (1.15, 1.86)**	1.24 (0.96, 1.62)	**1.36 (1.07, 1.71)**	1.26 (0.90, 1.77)	0.98 (0.76, 1.24)	**2.37 (1.73, 3.25)**	**2.50 (1.74, 3.60)**	**1.40 (1.07, 1.83)**	**0.77 (0.63, 0.94)**
1.31 (1.07, 1.59)	1.32 (0.99, 1.77)	**PEM+CT**	0.85 (0.68, 1.05)	0.93 (0.78, 1.11)	0.86 (0.64, 1.17)	**0.67 (0.55, 0.81)**	**1.62 (1.23, 2.14)**	**1.71 (1.22, 2.38)**	**0.96 (0.77, 1.19)**	**0.53 (0.46, 0.60)**
1.01 (0.81, 1.27)	1.03 (0.75, 1.40)	**0.78 (0.61, 0.98)**	**NIV+CT**	1.09 (0.89, 1.34)	1.02 (0.73, 1.41)	**0.78 (0.63, 0.98)**	**1.91 (1.42, 2.57)**	**2.01 (1.42, 2.86)**	1.13 (0.88, 1.44)	**0.62 (0.52, 0.73)**
1.05 (0.87, 1.27)	1.06 (0.80, 1.41)	0.80 (0.66, 0.98)	1.03 (0.82, 1.30)	**ATE+CT**	0.93 (0.69, 1.26)	**0.72 (0.60, 0.86)**	**1.75 (1.39, 2.20)**	**1.84 (1.37, 2.47)**	1.03 (0.89, 1.20)	**0.57 (0.50, 0.64)**
1.14 (0.80, 1.63)	1.16 (0.76, 1.75)	0.87 (0.61, 1.25)	1.13 (0.77, 1.64)	1.09 (0.76, 1.56)	**CAM+CT**	0.77 (0.57, 1.05)	**1.88 (1.30, 2.72)**	**1.98 (1.31, 3.00)**	1.11 (0.80, 1.54)	**0.61 (0.46, 0.8)**
1.12 (0.93, 1.36)	1.14 (0.86, 1.51)	0.86 (0.71, 1.05)	1.11 (0.88, 1.39)	1.07 (0.89, 1.30)	0.99 (0.69, 1.41)	**NIV+IPI**	**2.43 (1.84, 3.22)**	**2.56 (1.83, 3.59)**	**1.43 (1.15, 1.80)**	**0.79 (0.69, 0.91)**
1.18 (0.89, 1.58)	1.20 (0.84, 1.71)	0.91 (0.68, 1.21)	1.17 (0.86, 1.60)	1.13 (0.90, 1.43)	1.04 (0.69, 1.58)	1.05 (0.79, 1.40)	**ATE+BEV+CT**	1.05 (0.78, 1.43)	**0.59 (0.50, 0.70)**	**0.32 (0.25, 0.41)**
1.12 (0.77, 1.62)	1.13 (0.74, 1.73)	0.85 (0.59, 1.24)	1.10 (0.74, 1.63)	1.06 (0.76, 1.48)	0.98 (0.61, 1.58)	0.99 (0.68, 1.44)	0.94 (0.67, 1.33)	**NIV+BEV+CT**	**0.56 (0.44, 0.72)**	**0.31 (0.23, 0.42)**
0.95 (0.76, 1.19)	0.96 (0.71, 1.31)	**0.73 (0.58, 0.92)**	0.94 (0.72, 1.21)	0.90 (0.78, 1.05)	0.83 (0.57, 1.21)	0.84 (0.67, 1.06)	**0.80 (0.67, 0.95)**	0.85 (0.63, 1.14)	**BEV+CT**	**0.55 (0.46, 0.66)**
**0.82 (0.72, 0.94)**	0.83 (0.65, 1.07)	**0.63 (0.54, 0.73)**	**0.81 (0.67, 0.97)**	**0.78 (0.68, 0.90)**	**0.72 (0.52, 1.00)**	**0.73 (0.64, 0.84)**	**0.69 (0.54, 0.89)**	0.74 (0.52, 1.04)	0.87 (0.72, 1.04)	**CT**

C

≥3AEs (upper triangle) / ORR (lower triangle)

**PEM**	**1.68 (1.03, 2.79)**	**0.25 (0.17, 0.36)**	**0.21 (0.14, 0.30)**	**0.18 (0.13, 0.26)**	**0.16 (0.10, 0.25)**	**0.36 (0.25, 0.51)**	**0.10 (0.07, 0.16)**	**0.15 (0.08, 0.25)**	**0.16 (0.11, 0.24)**	**0.31 (0.24, 0.41)**
1.17 (0.76, 1.82)	**ATE**	**0.15 (0.09, 0.24)**	**0.12 (0.07, 0.20)**	**0.11 (0.07, 0.17)**	**0.09 (0.05, 0.17)**	**0.21 (0.13, 0.35)**	**0.06 (0.04, 0.11)**	**0.09 (0.05, 0.17)**	**0.10 (0.06, 0.16)**	**0.19 (0.12, 0.28)**
**0.33 (0.23, 0.47)**	**0.28 (0.18, 0.44)**	**PEM+CT**	0.83 (0.56, 1.22)	0.74 (0.52, 1.03)	0.63 (0.39, 1.01)	**1.45 (1.01, 2.07)**	**0.42 (0.27, 0.64)**	0.59 (0.34, 1.02)	**0.64 (0.43, 0.95)**	1.26 (0.97, 1.63)
**0.42 (0.29, 0.62)**	**0.36 (0.22, 0.57)**	1.28 (0.86, 1.90)	**NIV+CT**	0.89 (0.62, 1.28)	0.76 (0.46, 1.26)	**1.75 (1.19, 2.56)**	**0.51 (0.33, 0.79)**	0.71 (0.41, 1.26)	0.78 (0.51, 1.18)	**1.52 (1.13, 2.05)**
**0.49 (0.35, 0.68)**	**0.41 (0.27, 0.63)**	**1.47 (1.05, 2.06)**	1.15 (0.79, 1.66)	**ATE+CT**	0.86 (0.54, 1.35)	**1.97 (1.42, 2.73)**	**0.57 (0.43, 0.75)**	0.80 (0.51, 1.26)	0.87 (0.68, 1.12)	**1.71 (1.38, 2.13)**
**0.44 (0.28, 0.71)**	**0.38 (0.22, 0.65)**	1.35 (0.83, 2.15)	1.05 (0.64, 1.72)	0.91 (0.58, 1.43)	**CAM+CT**	**2.30 (1.44, 3.69)**	0.67 (0.40, 1.12)	0.94 (0.50, 1.76)	1.02 (0.62, 1.69)	**2.00 (1.34, 3.00)**
0.81 (0.57, 1.15)	0.69 (0.44, 1.07)	**2.45 (1.71, 3.51)**	**1.91 (1.29, 2.84)**	**1.67 (1.19, 2.33)**	**1.82 (1.15, 2.92)**	**NIV+IPI**	**0.29 (0.19, 0.44)**	**0.41 (0.24, 0.70)**	**0.44 (0.30, 0.65)**	0.87 (0.68, 1.11)
**0.25 (0.16, 0.39)**	**0.22 (0.13, 0.36)**	0.76 (0.50, 1.18)	**0.60 (0.37, 0.94)**	**0.52 (0.39, 0.70)**	**0.57 (0.34, 0.97)**	**0.31 (0.20, 0.48)**	**ATE+BEV+CT**	1.41 (0.88, 2.24)	**1.53 (1.16, 2.02)**	**3.00 (2.15, 4.19)**
**0.28 (0.17, 0.48)**	**0.24 (0.13, 0.43)**	0.86 (0.51, 1.45)	0.67 (0.39, 1.16)	**0.58 (0.38, 0.90)**	0.64 (0.35, 1.17)	**0.35 (0.21, 0.59)**	1.12 (0.72, 1.76)	**NIV+BEV+CT**	1.09 (0.75, 1.59)	**2.13 (1.32, 3.44)**
**0.44 (0.30, 0.66)**	**0.38 (0.23, 0.61)**	1.34 (0.90, 2.00)	1.05 (0.68, 1.61)	0.91 (0.70, 1.19)	1.00 (0.61, 1.65)	**0.55 (0.37, 0.81)**	**1.75 (1.31, 2.36)**	**1.56 (1.11, 2.20)**	**BEV+CT**	**1.96 (1.46, 2.63)**
1.04 (0.81, 1.33)	0.89 (0.62, 1.28)	**3.15 (2.43, 4.08)**	**2.46 (1.83, 3.32)**	**2.14 (1.72, 2.68)**	**2.34 (1.58, 3.51)**	**1.29 (1.00, 1.65)**	**4.12 (2.91, 5.84)**	**3.67 (2.33, 5.76)**	**2.35 (1.73, 3.18)**	**CT**

D

2yOR (upper triangle) / 1yOR (lower triangle)

**PEM**	1.27 (0.83, 1.94)	0.96 (0.68, 1.35)	1.40 (0.95, 2.04)	1.11 (0.80, 1.54)		1.08 (0.76, 1.50)	0.87 (0.57, 1.34)	1.02 (0.62, 1.71)	1.24 (0.85, 1.82)	**1.67 (1.32, 2.11)**
1.13 (0.76, 1.70)	**ATE**	0.75 (0.49, 1.16)	1.10 (0.70, 1.75)	0.87 (0.58, 1.33)		0.84 (0.55, 1.03)	0.69 (0.42, 1.13)	0.81 (0.46, 1.43)	0.98 (0.62, 1.55)	1.31 (0.93, 1.87)
**0.61 (0.44, 0.84)**	**0.53 (0.35, 0.81)**	**PEM+CT**	1.46 (0.99, 2.15)	1.16 (0.83, 1.62)		1.12 (0.79, 1.59)	0.91 (0.60, 1.39)	1.07 (0.64, 1.79)	1.29 (0.87, 1.91)	**1.74 (1.36, 2.23)**
0.82 (0.57, 1.19)	0.72 (0.46, 1.13)	1.36 (0.93, 1.99)	**NIV+CT**	0.80 (0.55, 1.15)		0.77 (0.52, 1.13)	0.63 (0.40, 0.99)	0.73 (0.42, 1.27)	0.89 (0.58, 1.36)	1.19 (0.89, 1.61)
0.97 (0.71, 1.31)	0.85 (0.57, 1.26)	**1.59 (1.16, 2.20)**	1.17 (0.81, 1.69)	**ATE+CT**		0.97 (0.70, 1.34)	0.79 (0.59, 1.05)	0.92 (0.60, 1.42)	1.12 (0.85, 1.46)	**1.50 (1.20, 1.88)**
0.89 (0.54, 1.44)	0.78 (0.45, 1.35)	1.47 (0.89, 2.40)	1.08 (0.64, 1.82)	0.92 (0.57, 1.49)	**CAM+CT**					
0.95 (0.69, 1.31)	0.84 (0.56, 1.26)	**1.57 (1.12, 2.20)**	1.16 (0.79, 1.68)	0.99 (0.72, 1.35)	1.07 (0.66, 1.75)	**NIV+IPI**	0.81 (0.53, 1.24)	0.95 (0.57, 1.60)	1.15 (0.78, 1.70)	**1.55 (1.22, 1.98)**
0.83 (0.54, 1.26)	0.73 (0.45, 1.19)	1.37 (0.89, 2.10)	1.01 (0.63, 1.60)	0.86 (0.63, 1.16)	0.93 (0.54, 1.63)	0.87 (0.57, 1.33)	**ATE+BEV+CT**	1.17 (0.75, 1.83)	**1.42 (1.06, 1.90)**	**1.91 (1.35, 2.70)**
0.80 (0.45, 1.40)	0.70 (0.38, 1.30)	1.32 (0.74, 2.32)	0.97 (0.53, 1.76)	0.83 (0.50, 1.35)	0.90 (0.46, 1.76)	0.84 (0.47, 1.48)	0.96 (0.58, 1.60)	**NIV+BEV+CT**	1.21 (0.86, 1.69)	**1.63 (1.03, 2.56)**
1.06 (0.72, 1.55)	0.93 (0.59, 1.47)	**1.75 (1.17, 2.60)**	1.28 (0.83, 1.98)	1.10 (0.83, 1.44)	1.19 (0.70, 2.03)	1.11 (0.75, 1.64)	1.28 (0.94, 1.73)	1.33 (0.88, 2.00)	**BEV+CT**	1.35 (0.99, 1.82)
**1.32 (1.05, 1.64)**	1.16 (0.83, 1.61)	**2.17 (1.71, 2.77)**	**1.60 (1.19, 2.15)**	**1.36 (1.10, 1.69)**	1.48 (0.96, 2.28)	**1.38 (1.09, 1.75)**	**1.59 (1.11, 2.26)**	1.65 (0.99, 2.77)	1.24 (0.91, 1.71)	**CT**

E

1yPR

**PEM**										
0.48 (0.03, 8.22)	**ATE**									
0.38 (0.03, 4.42)	0.78 (0.07, 9.32)	**PEM+CT**								
0.48 (0.03, 8.03)	1 (0.06, 17.35)	1.29 (0.11, 15)	**NIV+CT**							
0.32 (0.03, 3.38)	0.67 (0.06, 7.15)	0.86 (0.12, 5.6)	0.67 (0.06, 6.99)	**ATE+CT**						
0.62 (0.04, 10.21)	1.3 (0.07, 22.99)	1.66 (0.14, 19.81)	1.29 (0.07, 21.19)	1.93 (0.17, 22.18)	**CAM+CT**					
0.46 (0.03, 7.85)	0.97 (0.06, 17.07)	1.24 (0.11, 14.07)	0.96 (0.06, 15.87)	1.43 (0.14, 16.58)	0.75 (0.05, 12.8)	**NIV+IPI**				
0.13 (0.01, 2.08)	0.27 (0.01, 4.58)	0.34 (0.02, 3.86)	0.27 (0.01, 4.37)	0.4 (0.06, 2.48)	0.21 (0.01, 3.52)	0.28 (0.01, 4.6)	**ATE+BEV+CT**			
0.1 (0, 2.29)	0.21 (0.01, 4.92)	0.27 (0.01, 4.59)	0.21 (0.01, 4.99)	0.32 (0.02, 3.67)	0.16 (0.01, 3.92)	0.22 (0.01, 5)	0.8 (0.05, 12.88)	**NIV+BEV+CT**		
0.24 (0.02, 2.7)	0.5 (0.03, 5.81)	0.63 (0.06, 4.71)	0.49 (0.03, 5.79)	0.74 (0.13, 3.26)	0.38 (0.03, 4.54)	0.52 (0.03, 5.7)	1.87 (0.27, 12.14)	2.34 (0.31, 17.94)	**BEV+CT**	
1.07 (0.15, 7.73)	2.24 (0.29, 17.06)	2.85 (0.69, 11.85)	2.22 (0.3, 16.5)	3.32 (0.95, 12.43)	1.72 (0.23, 13.3)	2.31 (0.31, 16.61)	8.41 (1.12, 77.23)	10.48 (0.91, 162.02)	4.53 (1.04, 25.65)	**CT**

Supplementary Figure 11. Network meta-analysis of the first sensitive analysis excluding phase II trials. (A) Network diagrams for overall survival (OS) and progression-free survival (PFS) and 1-year OS rate and 1-year PFS rate (A1), 2-year OS rate (A2), objective response rate (ORR) (A3), adverse events of grade 3 or higher (≥ 3 AEs) (A4), OS and PFS of non-squamous subgroup(A5). Each circular node represents a type of treatment. Each line represents a type of head-to-head comparison. The size of the nodes and the thickness of the lines are weighted according to the number of studies evaluating each treatment and direct comparison, respectively. The total number of patients receiving a treatment was shown in brackets. (B) Pooled HR (95% CrIs) for OS and PFS. (C) Pooled OR (95% CrIs) for ≥ 3 AEs and ORR. (D) Pooled OR (95% CrIs) for 1-year OS rate and 2-year OS rate. (E) Pooled OR (95% CrIs) for 1-year PFS rate. Data in each cell are HR or OR (95% CrIs) for the comparison of row-defining treatment versus column-defining treatment. HR less than 1 and OR more than 1 favour upper-row treatment. Significant results are highlighted in red and bold. PEM: pembrolizumab; ATE: atezolizumab; NIV: nivolumab; DUR: durvalumab; TRE: tremelimumab; IPI: ipilimumab; CAM: camrelizumab; CEM: cemiplimab; BEV: bevacizumab; CT: chemotherapy; 1yOR: 1-year OS rate; 2yOR: 2-year OS rate; 1yPR: 1-year PFS rate

	Original result							Sensitivity analysis						
Treatment	OS	PFS	ORR	≥3AEs	1yPR	1yOR	2yOR	OS	PFS	ORR	≥3AEs	1yPR	1yOR	2yOR
PEM+CT	**0.933**	0.731	0.787	0.434	0.497	**0.961**	0.829	**0.917**	0.721	0.809	0.422	0.530	**0.962**	0.762
ATE+BEV+CT	0.724	0.937	**0.958**	**0.986**	0.886	0.669	**0.834**	0.750	0.937	**0.955**	**0.986**	0.847	0.683	**0.869**
NIV+IPI	0.678	0.249	0.282	0.215	0.438	0.500	0.618	0.663	0.249	0.282	0.215	0.447	0.482	0.582
CAM+CT	0.657	0.521	0.555	0.756	0.318	0.582	NA	0.646	0.516	0.577	0.759	0.344	0.566	NA
NIV+BEV+CT	0.582	**0.963**	0.908	0.798	**0.924**	0.669	0.572	0.610	**0.963**	0.887	0.800	**0.874**	0.694	0.645
ATE+CT	0.488	0.601	0.484	0.627	0.604	0.452	0.512	0.494	0.601	0.485	0.627	0.581	0.453	0.522
NIV+CT	0.429	0.488	0.587	0.543	0.419	0.702	0.239	0.414	0.484	0.612	0.547	0.433	0.686	0.215
PEM	0.392	0.022	0.150	0.098	0.158	0.415	0.730	0.375	0.022	0.151	0.098	0.196	0.400	0.699
ATE	0.379	0.273	0.055	0.002	0.425	0.248	0.372	0.366	0.273	0.054	0.002	0.435	0.236	0.340
BEV+CT	0.215	0.637	0.621	0.752	0.740	0.263	0.264	0.245	0.656	0.575	0.753	0.688	0.302	0.339
CT	0.024	0.079	0.114	0.289	0.093	0.039	0.031	0.022	0.079	0.114	0.292	0.126	0.037	0.026

Supplementary Figure 12. Bayesian ranking profiles of comparable treatments on efficacy and safety in original results and the sensitive analysis. Percentage of the SUCRA in each cell indicates the probability of each treatment being ranked from first (high value) to last (low value) on survival indicators for overall population. PEM: pembrolizumab; ATE: atezolizumab; NIV: nivolumab; DUR: durvalumab; TRE: tremelimumab; IPI: ipilimumab; CAM: camrelizumab; CEM: cemiplimab; BEV: bevacizumab; CT: chemotherapy; OS: overall survival; PFS: progression-free survival; ORR: objective response rate; ⩾3AEs: grade ⩾3 adverse events; 1yOR: 1-year OS rate; 2yOR: 2-year OS rate; 1yPR: 1-year PFS rate

2 Supplementary Tables

Supplementary Table 1: Checklist of the PRISMA extension for network meta-analysis.

Section/topic	Item#	Checklist item*	Reported on page #
**TITLE**			
Title	1	Identify the report as a network meta-analysis based on integrated analysis.	1
**ABSTRACT**			
Structured summary	2	Provide a structured summary including, as applicable:<br>• Background: main objectives;<br>• Methods: data sources; study eligibility criteria, participants, and interventions; main outcomes.<br>• Findings: number of studies and participants identified; summary estimates with corresponding confidence/credible intervals; treatment rankings.<br>• Interpretation: conclusions and implications of findings.<br>• Other: primary source of funding.	1-2
**INTRODUCTION**			
Rationale	3	Describe the rationale for the review in the context of what is already known.	2
Objectives	4	Provide an explicit statement of questions being addressed with reference to participants, interventions, comparisons, outcomes, and study design (PICOS).	2
**METHODS**			
Protocol and registration	5	Indicate if a review protocol exists, if and where it can be accessed (e.g., Web address), and, if available, provide registration information including registration number.	2
Eligibility criteria	6	Specify study characteristics and report characteristics (e.g., language, publication status) used as criteria for eligibility, giving rationale.	3
Information sources	7	Describe all information sources (e.g., databases with dates of coverage) in the search and date last searched.	3
Search	8	Present full electronic search strategy for at least one database, including any limits used, such that it could be repeated.	Supplementary Materials page 25-26
Study selection	9	State the process for selecting studies (i.e., screening, eligibility, included in the integrated analysis and network meta-analysis).	Figure 1
Data collection process	10	Describe method of data extraction from reports (e.g., piloted forms, independently, in duplicate) and any processes for obtaining and confirming data from investigators.	3
Data items	11	List and define all variables for which data were sought (e.g., PICOS, funding sources) and any assumptions and simplifications made.	3
Geometry of the network	S1	Describe methods used to explore the geometry of the treatment network under study and potential biases related to it. This should include how the evidence base has been graphically summarized for presentation, and what characteristics were compiled and used to describe the evidence base to readers.	3
Risk of bias in individual studies	12	Describe methods used for assessing risk of bias of individual studies (including specification of whether this was done at the study or outcome level), and how this information is to be used in any data synthesis.	3
Summary measures	13	State the principal summary measures.	3

Synthesis of results	14	Describe the methods of handling data and combining results of studies for each network meta-analysis.	3
Assessment of Inconsistency	S2	Describe the statistical methods used to evaluate the agreement of direct and indirect evidence in the treatment network(s) studied. Describe efforts taken to address its presence when found.	3
Risk of bias across studies	15	Specify any assessment of risk of bias that may affect the cumulative evidence.	3
Study characteristics	18	For each study, present characteristics for which data were extracted (e.g., study size, PICOS) and provide the citations.	3
Additional analyses	16	Describe methods of additional analyses, if done, indicating which were pre-specified. Sensitivity or subgroup analyses.	6
**RESULTS**			
Study selection	17	Give numbers of studies screened, assessed for eligibility, and included in the review, with reasons for exclusions at each stage, ideally with a flow diagram.	Figure 1
Presentation of network structure	S3	Provide a network graph of the included studies to enable visualization of the geometry of the treatment network	Figure 3
Summary of network geometry	S4	Provide a brief overview of characteristics of the treatment network. This may include commentary on the abundance of trials and randomized patients for the different interventions and pairwise comparisons in the network, gaps of evidence in the treatment network, and potential biases reflected by the network structure.	4
Risk of bias within studies	19	Present data on risk of bias of each study and, if available, any outcome level assessment.	Supplementary Materials page 2
Results of individual studies	20	For all outcomes considered (benefits or harms), present, for each study:1) simple summary data for each intervention group, and 2) effect estimates and confidence/credible intervals.	4
Synthesis of results	21	Present results of each meta-analysis done, including confidence/credible intervals. *In larger networks, authors may focus on comparisons versus a particular comparator (e.g. placebo or standard care), with full findings presented in an appendix. League tables and forest plots may be considered to summarize pairwise comparisons.* If additional summary measures were explored (such as treatment rankings), these should also be presented.	4-6
Exploration for inconsistency	S5	Describe results from investigations of inconsistency. This may include such information as measures of model fit to compare consistency and inconsistency models, *P* values from statistical tests, or summary of inconsistency estimates from different parts of the treatment network.	6
Risk of bias across studies	22	Present results of any assessment of risk of bias across studies.	6
Additional analysis	23	Give results of additional analyses, if done (e.g., sensitivity or subgroup analyses).	6

**DISCUSSION**			
Summary of evidence	24	Summarize the main findings including the strength of evidence for each main outcome; consider their relevance to key groups (e.g., healthcare providers, users, and policy makers).	7
Limitations	25	Discuss limitations at study and outcome level.	8
Conclusions	26	Provide a general interpretation of the results in the context of other evidence, and implications for future research.	8
**FUNDING**			
Funding	27	Describe sources of funding for the systematic review and other support.	8

PICOS = population, intervention, comparators, outcomes, study design.

*Text in italics indicates wording specific to reporting of network meta-analyses that has been added to guidance from the PRISMA statement.

† Authors may wish to plan for use of appendices to present all relevant information in full detail for items in this sectio

Supplementary Table 2: Literature search criteria in PubMed, Embase, the Cochrane Central Register of Controlled Trials.

PubMed

Search: (((((((((((((((((((((Bevacizumab[Title/Abstract]) OR (Aflibercept[Title/Abstract])) OR (sorafenib[Title/Abstract])) OR (sunitinib[Title/Abstract])) OR (axitinib[Title/Abstract])) OR (regorafenib[Title/Abstract])) OR (pazopanib[Title/Abstract])) OR (vandetanib[Title/Abstract])) OR (cabozantinib[Title/Abstract])) OR (Lenvatinib[Title/Abstract])) OR (Ramucirumab[Title/Abstract])) OR (Cediranib[Title/Abstract])) OR (Cilengitide[Title/Abstract])) OR (trebananib[Title/Abstract])) OR (dovitinib[Title/Abstract])) OR (Anlotinib[Title/Abstract])) OR ((((((("Angiogenesis Inhibitors"[MeSH Terms]) OR (vegfr[Title/Abstract])) OR (angiogenesis[Title/Abstract])) OR (angiogenic[Title/Abstract])) OR (angiostatic[Title/Abstract])) OR (angiogenetic[Title/Abstract])) OR (neovascularization[Title/Abstract]))) OR (((((((((((((Tislelizumab[Title/Abstract]) OR (toripalimab[Title/Abstract])) OR (camrelizumab[Title/Abstract])) OR (sintilimab[Title/Abstract])) OR (avelumab[Title/Abstract])) OR (durvalumab[Title/Abstract])) OR (atezolizumab[Title/Abstract])) OR (Nivolumab[Title/Abstract])) OR (pembrolizumab[Title/Abstract])) OR (BMS936559[Title/Abstract])) OR (Pidilizumab[Title/Abstract])) OR (Ipilimumab[Title/Abstract])) OR (Tremelimumab[Title/Abstract]))) OR (((((((((((((((((((((((("programmed death 1"[Title/Abstract]) OR (PD-1[Title/Abstract])) OR (PD1[Title/Abstract])) OR ("programmed death ligand 1"[Title/Abstract])) OR (PD-L1[Title/Abstract])) OR (PDL1[Title/Abstract])) OR ("PD L1"[Title/Abstract])) OR ("PD 1"[Title/Abstract])) OR (anti-PD-1[Title/Abstract])) OR (anti-PD-L1[Title/Abstract])) OR (Immunotherapy[MeSH Terms])) OR (Immunotherap*[Title/Abstract])) OR ("immune checkpoint"[Title/Abstract])) OR (ICB[Title/Abstract])) OR ("CTLA-4"[Title/Abstract])) OR ("CTLA 4"[Title/Abstract])) OR (CTLA4[Title/Abstract]) OR (LAG-3[Title/Abstract])) OR (LAG3[Title/Abstract])) OR ("LAG 3"[Title/Abstract])) OR (TIM-3[Title/Abstract])) OR (TIM3[Title/Abstract])) OR ("TIM 3"[Title/Abstract])) OR (TIGIT[Title/Abstract])) OR (VISTA[Title/Abstract]))))) AND (((((((Randomized Controlled Trial[Publication Type]) OR (controlled clinical trial[Publication Type])) OR ("Randomized Controlled Trial"[Title/Abstract])) OR ("controlled clinical trial"[Title/Abstract])) OR (randomized[Title/Abstract])) OR (randomised[Title/Abstract])) OR (randomly[Title/Abstract]))))) AND (((((NSCLC[Title/Abstract]) OR ("Non Small Cell"[Title/Abstract])) OR ("Non-Small-Cell"[Title/Abstract])) OR ("Non-Small Cell"[Title/Abstract])) OR ("Carcinoma, Non-Small-Cell Lung"[MeSH Terms]))) NOT (((((((((((((((((((((adjuvant[Title]) OR (Neoadjuvant[Title])) OR (second[Title])) OR (third[Title])) OR (Resection[Title])) OR (surgery[Title])) OR (chemoradiotherapy[Title])) OR (chemoradiation[Title])) OR (Microwave[Title])) OR ("early stage"[Title])) OR (radiotherapy[Title])) OR ("previous treated"[Title])) OR (EGFR-positive[Title])) OR (ALK-positive[Title])) OR (EGFR-mutan*[Title])) OR (EGFR-mutated[Title])) OR (ALK-mutan*[Title])) OR (ALK-mutated[Title])) OR (Gefitinib[Title])) OR (Erlotinib[Title])) OR (Osimertinib[Title])) Filters: from 2005 - 2020

Embase

#1 'non small cell lung cancer'/exp OR nsclc:ab,ti OR 'non small cell':ab,ti OR 'non-small-cell':ab,ti OR 'non-small cell':ab,ti

#2 'immunotherapy'/exp OR immunotherap*:ab,ti OR 'immune checkpoint':ab,ti OR icb:ab,ti OR 'programmed death 1 receptor'/exp OR pd1:ab,ti OR 'pd 1':ab,ti OR 'programmed death 1':ab,ti OR 'programmed death 1 ligand 1'/exp OR pdl1:ab,ti OR 'pd l1':ab,ti OR 'programmed death 1 ligand 1':ab,ti OR 'anti pd 1':ab,ti OR 'anti pd l1':ab,ti OR 'cytotoxic t lymphocyte antigen 4'/exp OR 'ctla 4':ab,ti OR ctla4:ab,ti OR lag3:ab,ti OR 'lag 3':ab,ti OR tim3:ab,ti OR 'tim 3':ab,ti OR tigit:ab,ti OR vista:ab,ti OR tislelizumab:ab,ti OR toripalimab:ab,ti OR camrelizumab:ab,ti OR sintilimab:ab,ti OR avelumab:ab,ti OR durvalumab:ab,ti OR atezolizumab:ab,ti OR nivolumab:ab,ti OR pembrolizumab:ab,ti OR bms936559:ab,ti OR pidilizumab:ab,ti OR ipilimumab:ab,ti OR tremelimumab:ab,ti

#3 'angiogenesis inhibitor'/exp OR vegfr:ab,ti OR angiogenesis:ab,ti OR angiogenic:ab,ti OR angiostatic:ab,ti OR angiogenetic:ab,ti OR neovascularization:ab,ti OR bevacizumab:ab,ti OR aflibercept:ab,ti OR sorafenib:ab,ti OR sunitinib:ab,ti OR axitinib:ab,ti OR regorafenib:ab,ti OR pazopanib:ab,ti OR vandetanib:ab,ti OR cabozantinib:ab,ti OR lenvatinib:ab,ti OR ramucirumab:ab,ti OR cediranib:ab,ti OR cilengitide:ab,ti OR trebananib:ab,ti OR dovitinib:ab,ti OR anlotinib:ab,ti

#4 #2 OR #3

#5 adjuvant:ti OR neoadjuvant:ti OR second:ti OR third:ti OR resection:ti OR surgery:ti OR chemoradiotherapy:ti OR chemoradiation:ti OR microwave:ti OR 'early stage':ti OR radiotherapy:ti OR 'previous treated':ti OR 'egfr positive':ti OR 'alk positive':ti OR 'egfr mutan*':ti OR 'egfr mutated':ti OR 'alk mutan*':ti OR 'alk mutated':ti OR gefitinib:ti OR erlotinib:ti OR osimertinib:ti

#6 #1 AND #4

#7 #6 NOT #5

#8 first:ab,ti OR 1st:ab,ti OR naive:ab,ti OR naïve:ab,ti OR untreated:ab,ti OR front:ab,ti OR 'no previous':ab,ti OR 1l:ab,ti OR 'newly diagnosed'

#9 #7 ADN #8 AND (2005:py OR 2006:py OR 2007:py OR 2008:py OR 2009:py OR 2010:py OR 2011:py OR 2012:py OR 2013:py OR 2014:py OR 2015:py OR 2016:py OR 2017:py OR 2018:py OR 2019:py OR 2020:py) AND 'randomized controlled trial'/de

Cochrane Central Register of Controlled Trials

#1 MeSH descriptor: [Carcinoma, Non-Small-Cell Lung] explode all trees

#2 ("Carcinoma, Non-Small-Cell Lung"):ti,ab,kw OR (NSCLC):ti,ab,kw OR ("Non Small Cell"):ti,ab,kw OR ("Non-Small-Cell"):ti,ab,kw OR ("Non-Small Cell"):ti,ab,kw

#3 #1 OR #2

#4 (Immunotherapy):ti,ab,kw OR (Immunotherap*):ti,ab,kw OR ("immune checkpoint"):ti,ab,kw OR (ICB):ti,ab,kw OR ("programmed death 1"):ti,ab,kw

#5 (PD-1):ti,ab,kw OR (PD1):ti,ab,kw OR ("programmed death ligand 1"):ti,ab,kw OR (PD-L1):ti,ab,kw OR (PDL1):ti,ab,kw

#6 ("PD L1"):ti,ab,kw OR ("PD 1"):ti,ab,kw OR (anti-PD-1):ti,ab,kw OR (anti-PD-L1):ti,ab,kw OR ("CTLA-4"):ti,ab,kw

#7 (CTLA4):ti,ab,kw OR ("CTLA 4"):ti,ab,kw OR (TIM-3):ti,ab,kw OR (TIM3):ti,ab,kw OR ("TIM 3"):ti,ab,kw

#8 (TIGIT):ti,ab,kw OR (VISTA):ti,ab,kw

#9 (Tislelizumab):ti,ab,kw OR (toripalimab):ti,ab,kw OR (camrelizumab):ti,ab,kw OR (sintilimab):ti,ab,kw OR (avelumab):ti,ab,kw

#10 (durvalumab):ti,ab,kw OR (atezolizumab):ti,ab,kw OR (Nivolumab):ti,ab,kw OR (pembrolizumab):ti,ab,kw OR (BMS936559):ti,ab,kw

#11 (Pidilizumab):ti,ab,kw OR (Ipilimumab):ti,ab,kw OR (Tremelimumab):ti,ab,kw

#12 MeSH descriptor: [Programmed Cell Death 1 Receptor] explode all trees

#13 MeSH descriptor: [CTLA-4 Antigen] explode all trees

#14 MeSH descriptor: [Immunotherapy] explode all trees

#15 #4 OR #5 OR #6 OR #7 OR #8 OR #9 OR #10 OR #11 OR #12 OR #13 OR #14

#16 MeSH descriptor: [Angiogenesis Inhibitors] explode all trees

#17 (vegfr):ti,ab,kw OR (angiogenetic):ti,ab,kw OR (angiogenic):ti,ab,kw OR (angiostatic):ti,ab,kw OR (angiogenesis):ti,ab,kw

#18 (neovascularization):ti,ab,kw OR (bevacizumab):ti,ab,kw OR (aflibercept):ti,ab,kw OR (sorafenib):ti,ab,kw OR (sunitinib):ti,ab,kw

#19 (axitinib):ti,ab,kw OR (regorafenib):ti,ab,kw OR (pazopanib):ti,ab,kw OR (vandetanib):ti,ab,kwOR (cabozantinib):ti,ab,kw

#20 (lenvatinib):ti,ab,kw OR (ramucirumab):ti,ab,kw OR (cediranib):ti,ab,kw OR (cilengitide):ti,ab,kw OR (trebananib):ti,ab,kw

#21 (dovitinib):ti,ab,kw OR (anlotinib):ti,ab,kw

#22 #16 OR #17 OR #18 OR #19 OR #20 OR #21

#23 #15 OR #22

#24 (Randomized Controlled Trial):pt OR (controlled clinical trial):pt OR ("Randomized Controlled Trial"):ti,ab,kw OR ("controlled clinical trial"):ti,ab,kw

#25 (randomized):ti,ab,kw OR (randomised):ti,ab,kw OR (randomly):ti,ab,kw

#26 #24 OR #25

#27 (first):ti,ab,kw OR (1st):ti,ab,kw OR (1L):ti,ab,kw OR (naive):ti,ab,kw OR (naïve):ti,ab,kw

#28 (untreated):ti,ab,kw OR (front):ti,ab,kw OR ("no previous"):ti,ab,kw OR (“newly diagnosed”)

#29 #27 OR #28

#30 #3 AND #23 AND #26 AND #29 with Cochrane Library publication date from Jan 2005 to Dec 2020, in Trials

Supplementary Table 3: RCTs for integrated analysis containing immune checkpoint inhibitor as 1st line treatments in patients with advanced wild-type non-small cell lung cancer.

Data are expressed as intervention/control unless indicated otherwise. Squ: squamous; Non-squ: non-squamous; NG: Not Given; CT: chemotherapy; Pd-CT: platinum-based doublet CT; PEM: pembrolizumab; pem: pemetrexed ; NIV: nivolumab; ATE: atelizumab; CEM: cemiplimab; CAB: carboplatin; BEV : bevacizumab; CIS: cisplatin; GEM: gemcitabine; PTX: paclitaxel; DUR: durvalumab; TRE: tremelimumab; IPI: ipilimumab; CAM: camrelizumab; M: maintenance therapy; BSC: best supportive care

Study (phase, ethnicity)	Author	Year	Population	Sample size	Male/ Female	Median ages	Intervention arm	Control arm
**Keynote 189** (Ⅲ)	Gandhi L	2018	non-squ	410/206	363/253	65/64	PEM (200 mg/3w)+pem (500 mg/m²/3w),+ CAB (AUC=5) /3w or CIS (75 mg/m²/3w), 4C +M (PEM+pem), 35C	pem (500 mg/m²/3w),+ CAB (AUC=5) /3w or CIS (75 mg/m²/3w), 4C +Mpem, 35C
**Keynote 407** (Ⅲ)	Paz-Ares, L. G.	2018	squ	278/281	455/104	65/65	PEM (200mg/3w, 35C)+CAB (AUC=6, d1)/3w+PTX (200 mg/m² /3w d1) or nab-PTX (100mg/m² /3w d1,8,15) , 4C	CAB (AUC=6, d1)/3w+PTX (200 mg/m² /3w d1) or nab-PTX (100 mg/m² /3w d1,8,15), 4C
**IMpower 130** (Ⅲ)	West H	2019	non-squ	483/240	415/308	64/65	CAB (AUC=6)/3w+nab-PTX (100 mg/m²/w), 4 or 6C+ ATE (1200 mg/3w)+MATE (1200 mg/3w)	CAB (AUC=6)/3w+nab-PTX (100 mg/m²/w), 4C+ BSC or Mpem/3w
**Impower 150** (Ⅲ)	Socinski MA	2018	non-squ	359/338	425/267	63/63	ATE (1200 mg/3w)+ CAB (AUC=6)/3w +PTX (200 mg/m² /3w)+ BEV (15 mg/kg/3w), 4 or 6C	CAB (AUC=6)/3w +PTX (200 mg/m² /3w)+ BEV (15 mg/kg/3w), 4 or 6C
				350/338	NG/NG	NG/63	ATE (1200 mg/3w) + CAB (AUC=6)/3w +PTX (200 mg/m² /3w), 4 or 6C	CAB (AUC=6)/3w+PTX(200 mg/m² /3w)+ BEV (15 mg/kg/3w), 4 or 6C
**IMpower 110** (Ⅲ)	Spigel, D.	2019	squ/non-squ PD-L1≥1%	277/277	NG/NG	NG/NG	ATE (1200 mg/3w)	Pd-CT/3w, 4 or 6C
**IMpower 131** (Ⅲ)	Jotte, R. M.	2018	squ	343/340	557/126	65/65	ATE (1200 mg/3w)+ CAB (AUC=6)/3w + nab-PTX (100 mg/m² /w), 4 or 6C	CAB (AUC=6)/3w+ nab-PTX (100 mg/m² /w), 4 or 6C
**Keynote 024** (Ⅲ)	Reck M	2016	squ/non-squ PD-L1≥50%	154/151	187/118	65/66	PEM (200 mg/3w), 35C	Pd-CT/3w, 4 to 6C
**Keynote 042** (Ⅲ)	Mok, T. S. K.	2019	squ/non-squ PD-L1≥1%	637/637	902/372	NG/NG	PEM (200 mg/3w), 35C	Pd-CT/3w, 4 to 6C
**CheckMate 9LA** (Ⅲ)	Reck M	2020	squ/non-squ	361/358	503/216	65/65	NIV (360mg/3w) + IPI (1mg/kg/6w) + Pd-CT/3w, 2C	Pd-CT/3w, 4C
**CCTG BR.34** (Ⅲ, Caucasian)	Natasha B. L.	2020	squ/non-squ	151/150	162/139	65/63	DUR (1500 mg/3w)+TRE (75mg/3w, 4C) + Pd-CT/3w, 4C+MDUR (1500 mg) alone (squ) or with Mpem (500mg/m² /4w) (non-squ)	DUR (1500 mg/4w) +TRE (75mg/4w, 4C) +MDUR (1500 mg)
**Checkmate 026** (Ⅲ)	Carbone DP	2017	squ/non-squ PD-L1≥1%	271/270	332/209	63/65	NIV (3 mg/kg/2w)	Pd-CT/3w , 6C
**Mystic** (Ⅲ)	Rizvi, N. A.	2018	squ/non-squ	374/372	506/240	65/64	DUR (20 mg/kg/4w)	Pd-CT, 4 to 6C
				372/372	516/228	66/64	DUR (20 mg/kg/4w) +TRE (1 mg/kg/4w up to 4C)	Pd-CT, 4 to 6C
**CheckMate 227** (Ⅲ)	Hellmann MD	2019 Part1	squ/non-squ	583/583	778/388	64/64	NIV (3mg/kg/2w) +IPI (1mg/kg/6w)	Pd-CT/3w, 4C
		2019 Part2	squ/non-squ	377/378	528/227	63/64	NIV (360mg/3w) + Pd-CT/3w, 4C	Pd-CT/3w, 4C
**Lynch** (Ⅱ)	Lynch TJ	2012	squ/non-squ	68/66	98/36	61/62	PTX (175 mg/m² /3w)+ CAB (AUC=6) /3w, 2C followed by IPI (10 mg/kg /3w) + PTX (175 mg/m² /3w) + CAB(AUC=6)/3w, 4C	PTX (175 mg/m² /3w)+CAB (AUC=6)/3w, 6C
**Govindan** (Ⅲ)	Govindan R	2017	squ	388/361	635/114	64/64	IPI (10 mg/kg/3w starting at cycle 3)+PTX (175 mg/m² /3w)+CAB (AUC=6)/3w, 6C	PTX (175 mg/m² /3w)+CAB (AUC=6)/3w, 6C
**EMPOWER-Lung1** (Ⅲ)	Sezer.A	2020	squ/non-squ	356/342	606/92	63/64	CEM 350mg/3w	Investigator-determined CT
**2020ESMOLBA54** (Ⅲ)	Jong-Seok.L	2020	non-squ	275/275	411/139	66/66	NIV 350mg/3w+BEV(15mg/kg/3w)+CAB (AUC=6)/3w+PTX (200mg/m² /3w) 6C	BEV(15mg/kg/3w)+CAB (AUC=6)/3w+PTX (200mg/m² /3w) 6C
**IMpower 132** (Ⅲ)	PapadimitRV.	2018	non-squ	292/286	384/194	64/63	ATE (1200 mg/3w)+ CAB (AUC=6)/3w or CIS (75 mg/m²/3w)+pem (500 mg/m²/3w) ,4 or 6C + M (ATE +pem)	CAB (AUC=6)/3w or CIS (75 mg/m²/3w) +pem (500 mg/m²/3w), 4 or 6C + Mpem
**Camel** (Ⅲ，China)	Zhou, C.	2019	non-squ	205/207	295/117	59/61	CAM (200 mg/3w)+ CAB (AUC=5)/3w+ pem (500 mg/m² /3w), 4 to 6C+ M(CAM+pem)	CAB (AUC=5)/3w +pem (500 mg/m² /3w) , 4 to 6C+ Mpem
**Keynote 021G** (Ⅱ)	Langer CJ	2016	non-squ	60/63	48/75	63/63	PEM (200 mg/3w) + CAB(AUC=5)/3w + pem (500 mg/m²/3w), 4C +M (PEM+pem), 2 years	pem (500 mg/m²/3w)+ CAB (AUC=5)/3w, 4C+ Mpem

Supplementary Table 4: RCTs for network meta-analysis with long-term survival time of ITT population.

Data are expressed as intervention/control unless indicated otherwise.

Squ: squamous; Non-squ: non-squamous; NG: Not Given; CT: chemotherapy; Pd-CT: platinum-based doublet CT; PEM: pembrolizumab; pem: pemetrexed ; NIV: nivolumab; ATE: atelizumab; CEM: cemiplimab; CAB: carboplatin;

BEV : bevacizumab; CIS: cisplatin; GEM: gemcitabine; PTX: paclitaxel; DUR: durvalumab; TRE: tremelimumab; IPI: ipilimumab; CAM: camrelizumab; M: maintenance therapy; BSC: best supportive care

Study (phase, ethnicity)	Author	Year	Population	Sample size	Male/ Female	Median ages	Intervention arm	Control arm
**Keynote 021G (Ⅱ)**	Langer CJ	2016	non-squ	60/63	48/75	63/63	PEM (200 mg/3w) + CAB(AUC=5)/3w + pem (500 mg/m^2/3w), 4C +M (PEM+pem), 2 years	pem (500 mg/m^2/3w)+ CAB (AUC=5)/3w, 4C+ Mpem
**Keynote 189 (Ⅲ)**	Gandhi L	2018	non-squ	410/206	363/253	65/64	PEM (200 mg/3w)+pem (500 mg/ m^2/3w),+ CAB (AUC=5) /3w or CIS (75 mg/m^2/3w), 4C +M (PEM+pem), 35C	pem (500 mg/m^2/3w),+ CAB (AUC=5) /3w or CIS (75 mg/m^2/3w), 4C +Mpem, 35C
**Keynote 407 (Ⅲ)**	Paz-Ares, L. G.	2018	squ	278/281	455/104	65/65	PEM (200mg/3w, 35C)+CAB (AUC=6, d1)/3w+PTX (200 mg/m^2/3w d1) or nab-PTX (100mg/m^2/3w d1,8,15) , 4C	CAB (AUC=6, d1)/3w+PTX (200 mg/m^2/3w d1) or nab-PTX (100 mg/m^2/3w d1,8,15), 4C
**Camel (Ⅲ，China)**	Zhou, C.	2019	non-squ	205/207	295/117	59/61	CAM (200 mg/3w)+ CAB (AUC=5)/3w+ pem (500 mg/m^2/3w), 4 to 6C+ M(CAM+pem)	CAB (AUC=5)/3w +pem (500 mg/m^2/3w) , 4 to 6C+ Mpem
**2020ESMOLBA54**	Jong-Seo k Lee	2020	non-squ	275/275	411/139	66/66	NIV(360mg/3w)+CAB(AUC=6)/3w+PTX(200mg/m^2/3w)+BEV(15mg/kg/3w)	CAB(AUC=6)/3w+PTX(200mg/m^2/3w)+BEV(15mg/kg/3w)
**IMpower 130 (Ⅲ)**	West H	2019	non-squ	483/240	415/308	64/65	CAB (AUC=6)/3w+nab-PTX (100 mg/m^2/w), 4 or 6C+ ATE (1200 mg/3w)+MATE (1200 mg/3w)	CAB (AUC=6)/3w+nab-PTX (100 mg/m^2/w), 4C+ BSC or Mpem/3w
**IMpower 132 (Ⅲ)**	Papadimi tRV.	2018	non-squ	292/286	384/194	64/63	ATE (1200 mg/3w)+ CAB (AUC=6)/3w or CIS (75 mg/m^2/3w)+pem (500 mg/m^2/3w) ,4 or 6C + M (ATE +pem)	CAB (AUC=6)/3w or CIS (75 mg/m^2/3w) +pem (500 mg/m^2/3w), 4 or 6C + Mpem
**Impower 150 (Ⅲ)**	Socinski MA	2018	non-squ	359/338	425/267	63/63	ATE (1200 mg/3w)+ CAB (AUC=6)/3w +PTX (200 mg/m^2/3w)+ BEV (15 mg/kg/3w), 4 or 6C	CAB (AUC=6)/3w +PTX (200 mg/m^2/3w)+ BEV (15 mg/kg/3w), 4 or 6C
				350/338	NG/NG	NG/63	ATE (1200 mg/3w) + CAB (AUC=6)/3w +PTX (200 mg/m^2/3w), 4 or 6C	CAB (AUC=6)/3w+PTX(200 mg/m^2/3w)+ BEV (15 mg/kg/3w), 4 or 6C
**Beyond (Ⅲ，China)**	Zhou C	2015	non-squ	138/138	152/124	57/56	PTX (175 mg/m^2/3w)+CAB (AUC=6)/3w+BEV (15 mg/kg/3w), 6C	PTX (175 mg/m^2/3w)+CAB (AUC=6)/3w 6C
**Niho (Ⅱ，Japan)**	Niho S	2012	non-squ	117/58	115/65	61/60	CAB (AUC=6)/3w +PTX(200 mg/m^2/3w)+BEV (15 mg/kg/3w), 6C	CAB (AUC=6)/3w+PTX (200 mg/m^2/3w), 6C
**IMpower 110 (Ⅲ)**	Spigel, D.	2019	squ/non-squ PD-L1≥1%	277/277	NG/NG	NG/NG	ATE (1200 mg/3w)	Pd-CT/3w, 4 or 6C
**Keynote 042 (Ⅲ)**	Mok, T. S. K.	2019	squ/non-squ PD-L1≥1%	637/637	902/372	NG/NG	PEM (200 mg/3w), 35C	Pd-CT/3w, 4 to 6C
**CCTG BR.34 (Ⅲ, Caucasian)**	Natasha B. L.	2020	squ/non-squ	151/150	162/139	65/63	DUR (1500 mg/3w)+TRE (75mg/3w, 4C) + Pd-CT/3w, 4C+MDUR (1500 mg) alone (squ) or with Mpem (500mg/m^2/4w) (non-squ)	DUR (1500 mg/4w) +TRE (75mg/4w, 4C) +MDUR (1500 mg)
**CheckMate 227 (Ⅲ)**	Hellman n MD	2019 Part1	squ/non-squ	583/583	778/388	64/64	NIV (3mg/kg/2w) +IPI (1mg/kg/6w)	Pd-CT/3w, 4C
		2019 Part2	squ/non-squ	377/378	528/227	63/64	NIV (360mg/3w) + Pd-CT/3w, 4C	Pd-CT/3w, 4C

Supplementary Table 5: RCTs for network meta-analysis with long-term survival time of PD-L1≥50% (or TC/IC=3) population.

Data are expressed as intervention/control unless indicated otherwise.

Squ: squamous; Non-squ: non-squamous; NG: Not Given; CT: chemotherapy; Pd-CT: platinum-based doublet CT; PEM: pembrolizumab; pem: pemetrexed ; NIV: nivolumab; ATE: atelizumab; CEM: cemiplimab; CAB: carboplatin;

BEV : bevacizumab; CIS: cisplatin; GEM: gemcitabine; PTX: paclitaxel; DUR: durvalumab; TRE: tremelimumab; IPI: ipilimumab; CAM: camrelizumab; M: maintenance therapy; BSC: best supportive care

Study (phase, ethnicity)	Author	Year	Population	Sample size	Male/ Female	Median ages	Intervention arm	Control arm
**Keynote 189** (Ⅲ)	Gandhi L	2018	non-squ	410/206	363/253	65/64	PEM (200 mg/3w)+pem (500 mg/ m²/3w),+ CAB (AUC=5) /3w or CIS (75 mg/m²/3w), 4C +M (PEM+pem), 35C	pem (500 mg/m²/3w),+ CAB (AUC=5) /3w or CIS (75 mg/m²/3w), 4C +Mpem, 35C
**EMPOWER-Lung1**	A.Sezer	2020	squ/non-squ	356/354	606/104	63/64	Cemipllimab(350mg/3w)	4-6 cycle CT decided by investigator
**2020ESMOLBA54**	Jong-Seok Lee	2020	non-squ	275/275	411/139	66/66	NIV(360mg/3w)+CAB(AUC=6)/3w+PTX(200mg/m^2 /3w)+BEV(15mg/kg/3w)	CAB(AUC=6)/3w+PTX(200mg/m^2 /3w)+BEV(15mg/kg/3w)
**IMpower 130** (Ⅲ)	West H	2019	non-squ	483/240	415/308	64/65	CAB (AUC=6)/3w+nab-PTX (100 mg/m²/w), 4 or 6C+ ATE (1200 mg/3w)+MATE (1200 mg/3w)	CAB (AUC=6)/3w+nab-PTX (100 mg/m²/w), 4C+ BSC or Mpem/3w
**Impower 150** (Ⅲ)	Socinski MA	2018	non-squ	359/338	425/267	63/63	ATE (1200 mg/3w)+ CAB (AUC=6)/3w +PTX (200 mg/m^2 /3w)+ BEV (15 mg/kg/3w), 4 or 6C	CAB (AUC=6)/3w +PTX (200 mg/m^2 /3w)+ BEV (15 mg/kg/3w), 4 or 6C
				350/338	NG/NG	NG/63	ATE (1200 mg/3w) + CAB (AUC=6)/3w +PTX (200 mg/m^2 /3w), 4 or 6C	CAB (AUC=6)/3w+PTX(200 mg/m^2 /3w)+ BEV (15 mg/kg/3w), 4 or 6C
**IMpower 110** (Ⅲ)	Spigel, D.	2019	squ/non-squ PD-L1≥1%	277/277	NG/NG	NG/NG	ATE (1200 mg/3w)	Pd-CT/3w, 4 or 6C
**IMpower 131** (Ⅲ)	Jotte, R. M.	2018	squ	343/340	557/126	65/65	ATE (1200 mg/3w)+ CAB (AUC=6)/3w + nab-PTX (100 mg/m^2 /w), 4 or 6C	CAB (AUC=6)/3w+ nab-PTX (100 mg/m^2 /w), 4 or 6C
**Keynote 024** (Ⅲ)	Reck M	2016	squ/non-squ PD-L1≥50%	154/151	187/118	65/66	PEM (200 mg/3w), 35C	Pd-CT/3w, 4 to 6C
**Keynote 042** (Ⅲ)	Mok, T. S. K.	2019	squ/non-squ PD-L1≥1%	637/637	902/372	NG/NG	PEM (200 mg/3w), 35C	Pd-CT/3w, 4 to 6C
**CheckMate 9LA** (Ⅲ)	Reck M	2020	squ/non-squ	361/358	503/216	65/65	NIV (360mg/3w) + IPI (1mg/kg/6w) + Pd-CT/3w, 2C	Pd-CT/3w, 4C
**Mystic** (Ⅲ)	Rizvi, N. A.	2018	squ/non-squ	374/372	506/240	65/64	DUR (20 mg/kg/4w)	Pd-CT, 4 to 6C
				372/372	516/228	66/64	DUR (20 mg/kg/4w) +TRE (1 mg/kg/4w up to 4C)	Pd-CT, 4 to 6C
**CheckMate 227** (Ⅲ)	Hellmann MD	2019 Part1	squ/non-squPD-L1≥1%	396/397	515/278	64/64	NIV (3mg/kg/2w) +IPI (1mg/kg/6w)	Pd-CT/3w, 4C
				396/397	532/261	64/64	NIV (240mg/2w)	Pd-CT/3w, 4C
**IMpower 132** (Ⅲ)	Papadimit RV.	2018	non-squ	292/286	384/194	64/63	ATE (1200 mg/3w)+ CAB (AUC=6)/3w or CIS (75 mg/m²/3w)+pem (500 mg/m²/3w) ,4 or 6C + M (ATE +pem)	CAB (AUC=6)/3w or CIS (75 mg/m²/3w) +pem (500 mg/m²/3w), 4 or 6C + Mpem

Supplementary Table 6: Pooled survival outcomes from integrated analysis of subgroups for the median overall survival time (POS)、1-year OS rate (P1OR) and 2-year OS rate (P2OR).

	POS	P1OR	P2OR
Overall population	16.20(14.79,17.60)	0.63(0.59,0.66)	0.37(0.33,0.41)
Exclude MYSTIC/Govindan/Lynch	17.32(16.16,18.47)	0.65(0.62,0.68)	0.40(0.35,0.44)
Exclude Govindan/Lynch	16.64(15.12,18.15)	0.63(0.60,0.67)	0.39(0.35,0.43)
anti-PD-1	18.00(15.52,20.48)	0.67(0.64,0.72)	0.43(0.36,0.50)
anti-PD-L1	17.23(15.17,19.30)	0.59(0.54,0.63)	0.33(0.28,0.39)
anti-CTLA-4	12.92(11.78,14.06)	0.54(0.49,0.68)	0.22(0.16,0.28)
anti-PD-1/L1+anti-CTLA-4	14.83(12.11,17.56)	0.57(0.51,0.64)	0.34(0.26,0.42)
PEM-containg	19.09(15.69,22.48)	0.67(0.61,0.73)	0.46(0.39,0.53)
ATE-containg	17.23(15.17,19.30)	0.60(0.57,0.64)	0.34(0.28,0.41)
IPI-containg	13.10(11.80,14.39)	0.54(0.49,0.58)	0.22(0.16,0.28)
NIV-containg	16.36(12.54,20.18)	0.62(0.51,0.73)	0.31(0.15,0.46)
DUR+TRE-containg	13.75(10.22,17.27)	0.54(0.45,0.62)	0.29(0.18,0.39)
NIV+IPI-containg	16.54(14.68,18.40)	0.62(0.59,0.65)	0.41(0.38,0.44)
CEM	NA	0.70(0.65,0.75)	0.49(0.43,0.54)
DUR	12.30(9.90,14.70)	0.51(0.46,0.56)	0.29(0.24,0.33)
CAM+CT	NA	0.75(0.69,0.81)	NA
ATE+BEV+CT	19.50(16.90,22.10)	0.67(0.63,0.72)	0.43(0.38,0.49)
NIV+BEV+CT	NA	0.81(0.76,0.85)	0.55(0.49,0.61)
PEM	19.85(10.60,29.10)	0.64(0.52,0.76)	0.44(0.33,0.56)
NIV	14.40(11.55,17.25)	0.56(0.50,0.62)	0.23(0.18,0.28)
ATE	17.50(12.35,22.65)	0.58(0.52,0.64)	0.38(0.32,0.44)
PEM+CT	19.54(14.73,24.34)	0.69(0.64,0.75)	0.49(0.36,0.61)
ATE+CT	17.22(18.43,19.16)	0.61(0.57,0.65)	0.34(0.26,0.41)
IPI+CT	13.10(11.80,14.39)	0.54(0.49,0.58)	0.22(0.16,0.28)
NIV+CT	18.30(15.50,21.10)	0.67(0.62,0.72)	0.38(0.34,0.43)
DUR+TRE	12.11(9.47,14.76)	0.50(0.43,0.58)	0.30(0.25,0.34)
NIV+IPI	17.10(14.75,19.45)	0.62(0.58,0.66)	0.40(0.36,0.44)
DUR+TRE+CT	16.60(13.35,19.85)	0.60(0.52,0.68)	0.28(0.08,0.49)
NIV+IPI+CT	15.60(12.55,18.65)	0.63(0.58,0.68)	0.43(0.38,0.48)

	Subgroups	P1OR	P2OR
PDL1≥50%	anti-PD-1	0.68(0.65,0.70)	0.47(0.45,0.50)
	anti-PD-L1	0.63(0.60,0.67)	0.41(0.37,0.45)
	anti-PD-1/L1+anti-CTLA-4	0.68(0.62,0.73)	0.48(0.42,0.53)
	ICIs+BEV+CT	0.75(0.65,0.85)	0.56(0.45,0.67)
	single-ICI	0.67(0.64,0.69)	0.47(0.44,0.50)
	single-ICI+CT	0.66(0.62,0.69)	0.43(0.39,0.47)
	dual-ICIs	0.67(0.60,0.73)	0.48(0.41,0.55)
	dual-ICIs+CT	0.70(0.59,0.80)	047(0.36,0.58)

Supplementary Table 7: Comparisons of the fit of consistency and inconsistency models using deviance information criteria (DIC).

		model		
		consistency,fixed	consistency,random	inconsistency
overall	OS	27.00	27.37	27.08
	PFS	28.51	28.21	27.21
	ORR	54.99	55.25	52.13
	≥3AEs	51.61	52.41	48.92
	1yOR	52.59	53.97	53.77
	2yOR	52.73	52.37	53.49
	1yPR	63.34	**57.20**	53.16
PDL1≥50 or TC/IC=3	OS	20.94	22.00	20.93
	PFS	24.36	**17.81**	17.78
	1yOR	34.51	35.97	34.52
	2yOR	34.60	36.14	34.33
	1yPR	39.71	**34.05**	33.83
high-TMB	OS	16.15	16.87	16.16
	PFS	14.00	14.02	14.00
non-squamous	OS	20.24	21.33	20.34
	PFS	21.32	20.07	20.94
squamous	OS	4.00	4.00	3.99
	PFS	3.99	4.00	4.00

Supplementary Table 8: Node-splitting analysis of inconsistency.

Nodes	Direct effect	Indirect effect	Overall	P
**Overall survival**				
BEV+CT,ATE+CT	1.20(1.00,1.40)	0.97(0.72,1.30)	1.10(0.98,1.30)	0.17
CT,ATE+CT	1.20(1.10,1.40)	1.50(1.10,2.10)	1.30(1.10,1.40)	0.17
CT,BEV+CT	1.30(1.00,1.70)	1.00(0.81,1.30)	1.10(0.96,1.30)	0.17
**Progression-free survival**				
BEV+CT,ATE+CT	1.10(0.90,1.30)	0.79(0.61,1.00)	0.98(0.85,1.10)	0.07
CT,ATE+CT	1.70(1.50,1.90)	2.20(1.70,3.00)	1.70(1.60,2.00)	0.07
CT,BEV+CT	2.10(1.70,2.70)	1.60(1.30,1.90)	1.80(1.50,2.10)	0.07
**Objective response rate**				
BEV+CT,ATE+CT	0.96(0.71,1.30)	1.80(1.10,2.90)	1.20(0.89,1.50)	**0.03**
CT,ATE+CT	0.51(0.40,0.64)	0.27(0.16,0.45)	0.45(0.36,0.56)	**0.03**
CT,BEV+CT	0.28(0.19,0.42)	0.53(0.36,0.78)	0.39(0.30,0.52)	**0.03**
**Grade ≥3 adverse events**				
BEV+CT,ATE+CT	1.30(0.99,1.70)	0.67(0.38,1.20)	1.10(0.89,1.50)	**0.03**
CT,ATE+CT	0.53(0.42,0.67)	1.00(0.59,1.80)	0.58(0.47,0.73)	**0.03**
CT,BEV+CT	0.79(0.48,1.30)	0.40(0.28,0.58)	0.51(0.38,0.69)	**0.03**
**1-year overall survival rate**				
BEV+CT,ATE+CT	0.82(0.60,1.10)	1.10(0.67,1.80)	0.89(0.69,1.20)	0.35
CT,ATE+CT	0.78(0.62,0.98)	0.59(0.35,1.00)	0.74(0.60,0.92)	0.35
CT,BEV+CT	0.72(0.47,1.10)	0.94(0.64,1.40)	0.83(0.62,1.10)	0.35
**2-year overall survival rate**				
BEV+CT,ATE+CT	0.79(0.58,1.10)	1.00(0.64,1.60)	0.85(0.66,1.10)	0.40
CT,ATE+CT	0.72(0.56,0.91)	0.57(0.35,0.93)	0.69(0.55,0.85)	0.40
CT,BEV+CT	0.72(0.49,1.00)	0.91(0.61,1.30)	0.80(0.61,1.10)	0.40
**1-year progression-free survival rate**				
BEV+CT,ATE+CT	0.79(0.38,1.60)	3.90(1.40,11.00)	1.40(0.53,4.70)	**0.02**
CT,ATE+CT	0.40(0.24,0.68)	0.08(0.03,0.25)	0.29(0.11,0.71)	**0.02**
CT,BEV+CT	0.10(0.04,0.24)	0.51(0.21,1.20)	0.21,0.06,0.50)	**0.02**
**Overall survival for non-squ**				
BEV+CT,ATE+CT	1.20(1.00,1.40)	0.97(0.70,1.30)	1.10(0.98,1.30)	0.17
CT,ATE+CT	1.20(1.10,1.40)	1.50(1.10,2.10)	1.30(1.10,1.40)	0.17
CT,BEV+CT	1.30(1.00,1.70)	1.00(0.81,1.30)	1.10(0.96,1.30)	0.17
**Progression-free survival for non-squ**				
BEV+CT,ATE+CT	1.10(0.90,1.30)	0.79(0.61,1.00)	0.98(0.85,1.10)	0.07
CT,ATE+CT	1.70(1.50,1.90)	2.20(1.70,3.00)	1.70(1.60,2.00)	0.07
CT,BEV+CT	2.10(1.70,2.70)	1.60(1.30,1.90)	1.80(1.50,2.10)	0.07
